# Translocation of promoter-conserved hatching enzyme genes with intron-loss provides a new insight in the role of retrocopy during teleostean evolution

**DOI:** 10.1038/s41598-019-38693-6

**Published:** 2019-02-21

**Authors:** Tatsuki Nagasawa, Mari Kawaguchi, Tohru Yano, Sho Isoyama, Shigeki Yasumasu, Masataka Okabe

**Affiliations:** 1 Department of Anatomy, The Jikei University School of Medicine, 3-25-8 Nishishimbashi, Minato-ku, Tokyo 105-8461 Japan; 2 Research Fellow of the Japan Society for the Promotion of Science (JSPS), Tokyo, 102-0083 Japan; 3 Department of Materials and Life Sciences, Faculty of Science and Technology, Sophia University, 7-1 Kioi-cho, Chiyoda-ku, Tokyo 102-8554 Japan

## Abstract

The hatcing enzyme gene (*HE*) encodes a protease that is indispensable for the hatching process and is conserved during vertebrate evolution. During teleostean evolution, it is known that *HE* experienced a drastic transfiguration of gene structure, namely, losing all of its introns. However, these facts are contradiction with each other, since intron-less genes typically lose their original promoter because of duplication via mature mRNA, called retrocopy. Here, using a comparative genomic assay, we showed that *HEs* have changed their genomic location several times, with the evolutionary timings of these translocations being identical to those of intron-loss. We further showed that *HEs* maintain the promoter sequence upstream of them after translocation. Therefore, teleostean *HE*s are unique genes which have changed intra- (exon-intron) and extra-genomic structure (genomic loci) several times, although their indispensability for the reproductive process of hatching implies that *HE* genes are translocated by retrocopy with their promoter sequence.

## Introduction

During vertebrate evolution, some vertebrates moved to the land, whereas the ancestors of teleosts remained underwater where it prospered, as indicated by this group now constituting half of the extant vertebrate species^1,2^. However, such species are constantly exposed to mechanical stresses, such as water flow and collisions with pebbles, underwater. Because embryos are particularly susceptible to external stresses, their ability to survive this period without difficulty is essential for their successful breeding in water. As is also found in teleosts, the embryos of most animals are protected by an architectural feature called the egg envelope. Acquiring a more robust egg envelope provides an effective protection during the embryonic period, but also acts as a barrier for the embryo to exit the egg during the hatching period. To enable hatching from a hard egg envelope, embryos secrete a protease that can digest egg envelope proteins, called the hatching enzymes (HEs).

*HE* genes have already been identified in a range of taxa from invertebrates (such as sea urchin and sea squirt) to vertebrates (except mammals)^3–5^, and they are considered to be indispensable for oviparous animals. In teleosts, *HE* genes have already been identified in over 40 species, and studies have revealed the homologous nature of all *HEs* identified thus far, which belong to the astacin superfamily^6–8^. Moreover, the developmental expression pattern of *HE* genes was found to be highly conserved in teleosts; specifically, such expression starts in a homologous cell population (anterior part of the hypoblast, called the “polster” or “pillow”) at the homologous developmental stage (at the late phase of gastrulation)^9–13^. In addition to these findings, considering that HEs are molecules that exclusively function during the hatching period^14^, it is expected that the regulatory mechanism of *HE* expression is highly conserved during teleostean evolution, although to the best of our knowledge, no promoter assay of teleostean *HEs* has yet been conducted.

**Figure 1:**
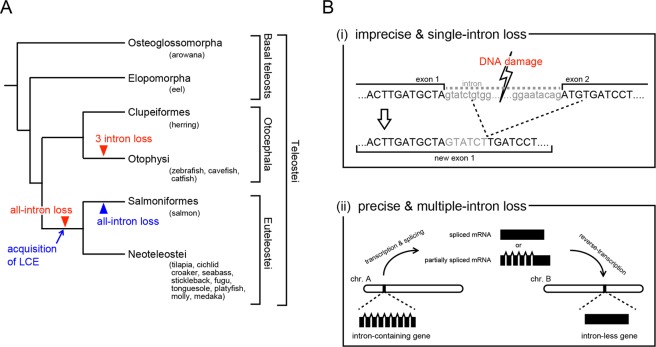
The schematic cartoon of intron-loss in teleostean *HEs*. (**A**) The history of intron-loss in teleostean *HEs*. Red and blue arrowheads indicate the evolutionary timing of multiple-intron loss in clade I and II genes, respectively. (**B**) Intron-loss mechanism. There are two types of intron-loss mechanisms; imprecise single-intron loss caused by DNA-repairing error (i), and precise multiple-intron loss by retrocopy (ii).

Although it would be expected that genomic structures around *HEs* (including the promoter) are conserved because the expression pattern is similar among teleostean species, it is actually known that the intra-genomic structures (exon–intron structures) of *HE*s have changed several times (e.g., intron loss; Fig. 1A)^13^. There are two different patterns of intron loss in *HEs*. In the first pattern, only one intron disappears, and several nucleotides are inserted or deleted. From sequence comparison among closely related species, this intron loss is thought to be caused by an error in DNA repair due to non-homologous end-joining or homologous recombination^11,14^ (Fig. 1Bi). In the second pattern, which is focused on this study, multiple (or all) introns are precisely removed without any insertion or deletion (Fig. 1Bii). In basal teleosts (osteoglossomorpha and elopomorpha), *HEs* comprise nine exons interrupted by eight introns^10,13^. Because this exon–intron structure is basically also conserved among tetrapods (frog, bird, and reptiles)^5^, it is considered that this structure is the ancestral type. *HEs* were subsequently duplicated, and these duplicated *HEs* are classified into clade I [e.g., *high choriolytic enzyme* (*HCE*)] and clade II [e.g., *low choriolytic enzyme* (*LCE*)] depending on their specificities of cleavage against egg envelope protein and molecular phylogenetic analysis^14–16^. During evolution, clade I genes independently lost multiple introns in separate lineages—three introns in the common ancestor of otophysi and all eight introns in the common ancestor of euteleostei. Moreover, clade II genes lost all eight introns in the common ancestor of salmoniformes and esociformes (Fig. 1A). In these events, introns were precisely lost without any additional insertion/deletion in the exonic regions^5,13^.

The exon–intron structures of homologous genes are generally considered to have been conserved during the course of evolution (even among different phyla)^17–19^. Therefore, it is thought to be unusual for intron-loss events to frequently occur in a taxon. However, it has also been reported that some genes lost one or more introns during the evolution of eukaryotes, which was considered to have occurred via retrocopy^20–23^. Retrocopy, also called as retroposition or retrotranslocation, is the phenomenon whereby a gene newly integrates its own cDNA into a different genomic location using an autonomous retrotransposon system. The reverse transcription of completely spliced mRNA (mature mRNA) causes intron-less genes, while that of intermediate mRNA produces genes with the loss of some introns, like in the case of otophysan *HEs*. The genes generated by retrocopy (retrocopied genes) precisely lose introns without any insertion/deletion because these genes are derived from a complementary strand of mRNA. Although it is known that the retrocopied genes translocate to different genomic loci, it is still unclear that whether the *HE* had experienced such translocation at the evolutionary timing of intron-loss or not. Basically, many of the retrocopied genes become intron-less-pseudogenes (processed pseudogenes) due to loss of the promoter sequence located upstream of the transcriptional region^24,25^. Although it has also been reported that some retrocopied genes survived by acquiring a new promoter sequence at a new location^26^, it is unlikely that *HEs* that display a conservative developmental expression pattern were changed to an intron-less state via conventional retrocopy.

Facilitated by the recent emergence of next-generation sequencers, high-quality genomic data are now available for many teleostei (cited in Methods). This has made it possible to comprehensively compare the genomic sequences around *HEs* among many teleost species. In this paper, we first describe the details of the evolutionary changes of the genomic location of *HEs* as determined by genome-scale synteny analysis, in addition to the exon–intron structure. Second, from promoter analysis, we showed that *HEs* maintains promoter sequences, although the *HEs* lose their all introns. From these results, we proposed the hypothesis of the mechanisms of intron-loss in teleostean *HEs*. Finally, we discuss the effects of this retrocopy system on the molecular evolution of *HEs* and the reproductive systems of teleosts.

## Results

### *In silico* cloning and phylogenetic analysis of HEs

First, to compare the genomic loci of *HEs* among teleostean species, we newly cloned *HEs* from Atlantic herring, channel catfish, large yellow croaker, European seabass, African cichlid, and Chinese red tonguesole *in silico*. These cloned sequences included six well-conserved cysteine residues with a role in the higher-order structure and two consensus sequences at the active site (HExxHxxGFxHExxRxDR and SxMHY, where “x” represents any residue) that are involved in catalytic activity (Fig. S1). In the tree produced by phylogenetic analysis, there were two large clades (clades I and II), as found in previous studies^13,14^. Atlantic herring *HEa*-*HEd*, channel catfish *HEa* and *HEb*, and euteleostean *HCEs* were included in clade I, whereas euteleostean *LCEs* were included in clade II (Fig. S2). The accession numbers of the sequences used for this analysis are listed in Table S1. In molly, in which breeding involves eggs being kept within the maternal body (ovoviviparous fish), full-length *HE* genes were not observed in the genome, whereas vestiges of the sequences of *HCE* (Fig. S3A,B) and *LCE* (Fig. S3C) were found. It is also known that *HEs* were pseudogenized in other ovoviviparous fishes, namely platyfish^27^ and black rockfish^28^. On the other hand, in some oviparous fishes, such as seabass (Fig. S4A), tilapia (Fig. S4B), and stickleback (Fig. S4C), vestiges of *HCE*-like sequences were found, in addition to full-length *HCEs*. The origins of these vestiges of *HCEs* in oviparous fishes are described later.

### Synteny analysis around the clade I genes

**Figure 2:**
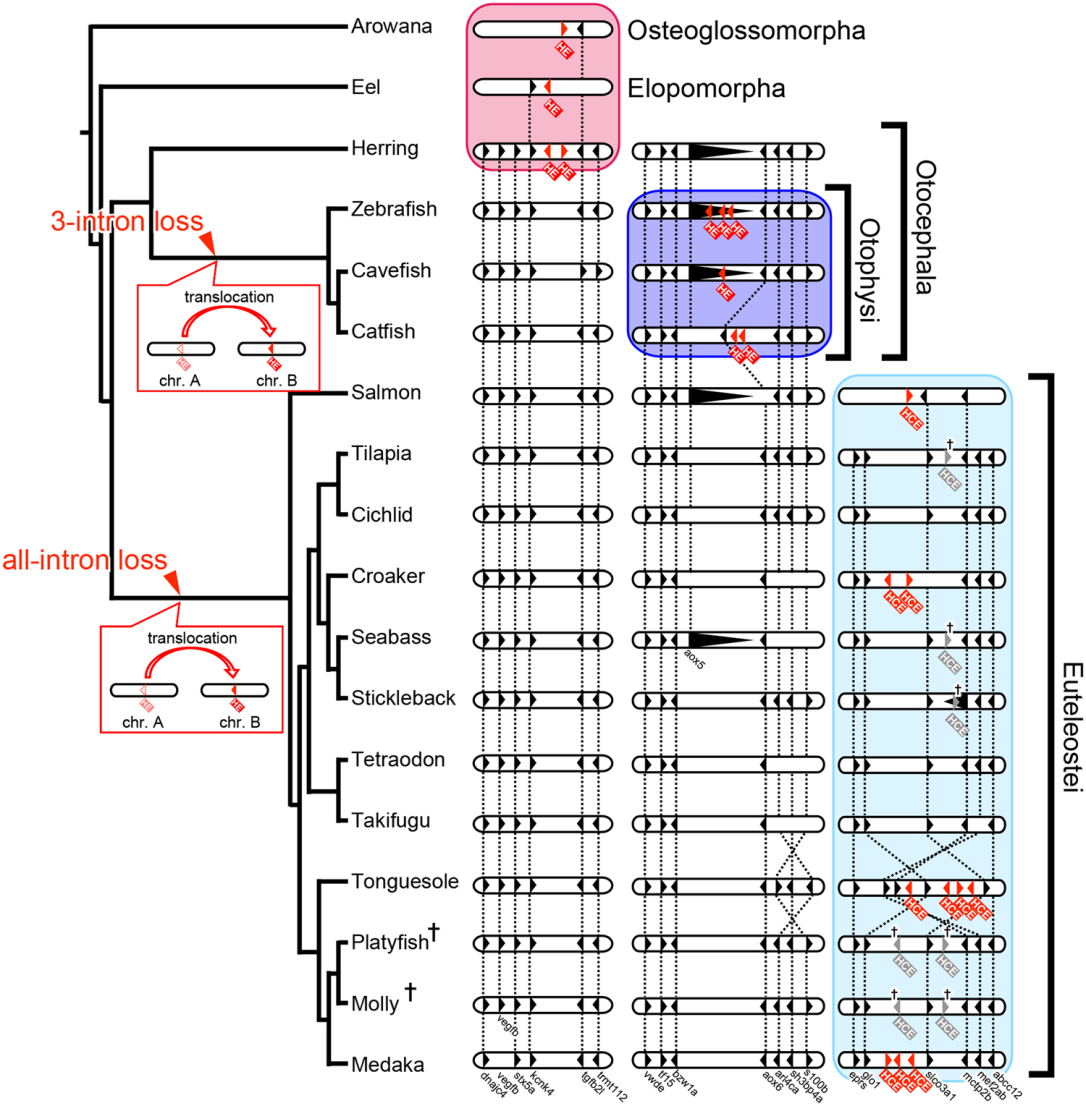
Putative evolutionary timing of the translocation and intron loss of clade I genes in teleostei. The orders of genes around clade I genes (in Fig. S5) are summarized, with reference to the teleostean evolutionary lineage. Oblongs and triangles indicate chromosomes and the coding regions of the genes, respectively. Red and gray triangles with a cross indicate the coding region and the vestiges of the clade I genes, respectively. The direction in which the triangles are pointing is the direction of transcription. The homologous genes are connected with broken lines. Each species is described using its common name. The crosses for platyfish and molly (ovoviviparous fishes) indicate that they do not have clade I genes in their genomes. A scheme of the evolutionary lineage is shown on the left. The putative timings of events of intron loss and translocation are indicated as red arrowhead and red balloon, respectively.

We next compared the genome synteny (the order of the neighboring genes) around the clade I genes among 18 teleostean species, and found that the genomic location of these genes varied among the lineages. The results also indicated that the putative evolutionary timing of the change of genomic location was completely consistent with the timing of intron loss (detailed results are shown in Fig. S5 and the overall outline is shown in Fig. 2).

First, we compared the genome synteny around *HEs* in arowana, eel, and herring, which are species that diverged at an early stage in teleostean evolution (Fig. S5A). In herring, *tgfb2l* and *kcnk4* were found to be arranged in a tail-to-tail orientation, and clade I *HEs* were located between them (red square in Fig. 2). Similarly, in arowana (which belongs to osteoglossomorpha) and eel (which belongs to elopomorpha), *HEs* were located downstream of *tgfb2l* or *kcnk4*, although the genome scaffold registered in the database was short in these species (red square in Fig. 2). These results suggest that the synteny around *HEs* in teleostei that diverged at an early stage is conserved. Interestingly, although the order of the neighboring genes was conserved, clade I genes (even just their vestiges) were not found at the corresponding region in the other species (below the red square in Fig. 2).

In contrast to the findings described above, the genome synteny analysis of clade I genes in zebrafish, cavefish, and catfish (which belong to the otophysi: lower taxon of otocephala) showed that these genes translocated to different genomic locations (Fig. S5B). For example, clade I genes of zebrafish and cavefish were located at the 14th intron of *aox5* and orientated in the opposite direction, whereas those of catfish were located in a similar location. Interestingly, the genome synteny around clade I *HEs* was highly conserved among all teleosts examined, with the only exception being for clade I *HEs* themselves. These results suggest that clade I genes translocated from their ancestral location (red square in Fig. 2) to a new location (blue square in Fig. 2) in a common ancestor of otophysi (upper balloon in Fig. 2); moreover, the evolutionary timing of this corresponded to an event in which three introns were lost (upper red arrowhead in Fig. 2).

A further syntenic analysis revealed that euteleostean clade I genes (also called “*HCE*” in euteleostei) were also translocated (Fig. S5C). In euteleostei, many *HCEs* adopted new syntenic positions that differed from their ancestral positions. For example, the order of three genes, *glo1*, *slco3a1*, and *mctp2b*, was basically conserved among euteleostei. In salmon, croaker, tonguesole, and medaka, *HCE* gene(s) were located around this genomic region. The vestiges of sequences of *HCEs* of the ovoviviparous platy and molly (shown in Fig. S3A,B) and those of the oviparous tilapia, seabass, and stickleback (shown in Fig. S4) were also located around the same location (gray triangles with a cross in Fig. S5C). Because many euteleostean species, including species of salmon that diverged at an early stage, have retained *HCEs* (or vestiges of them), it seems that this genomic location of *HCE* was the ancestral genomic location in euteleostei, suggesting that clade I genes translocated from their ancestral location in teleostei (red square in Fig. 2) to this location (light blue square in Fig. 2) and that the evolutionary timing of this (lower balloon in Fig. 2) corresponded to the timing of the loss of all introns (lower arrowhead in Fig. 2).

The consistency of the evolutionary timing of the translocation and intron loss, as identified in this analysis, suggests that the introns of clade I genes were lost by retrocopy. However, in general, retrocopy is caused by gene duplication via mRNA, but this is inconsistent with our findings that neither clade I genes nor their vestiges were found at their original position. We considered that the accumulation of mutations over the long intervening period caused the complete obliteration of any vestige of these genes.

### Independent translocation and vestiges of clade I genes in euteleostei

**Figure 3:**
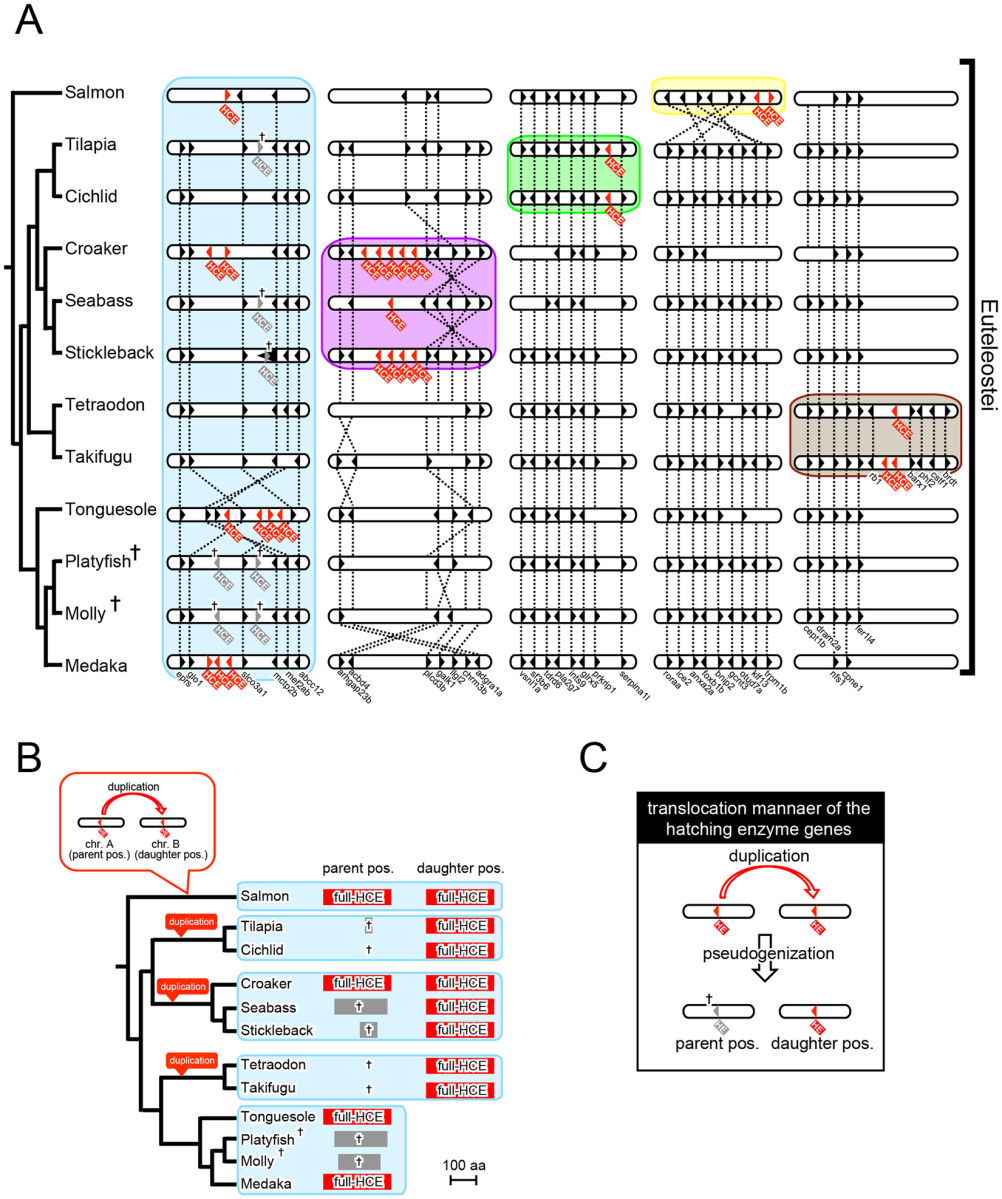
Translocation manner of *HEs* deduced from comparison of the *HCEs* (clade I genes) in euteleostei. (**A**) Putative evolutionary timing of the translocation and intron loss of *HCE*s. The orders of genes around *HCEs* (in Fig. S6) are summarized as in Fig. 1. The interior of the light blue box shows the same results as in Fig. 1. (**B**) A schematic diagram of the gene duplication from the ancestral position (parent pos.) to a new position (daughter position) and the pseudogenization of *HCEs* in euteleostei. The full-length *HCE* (full HCE in red rectangles) and the vestiges of *HCE* (crosses with/without gray rectangles) are summarized, referring to the genomic position and evolutionary lineages. The putative evolutionary timings of the gene duplication of *HCEs* are indicated as red balloons. (**C**) The putative mechanism of translocation of the *HEs*. Translocation of *HEs* occurred by gene duplication into another chromosome (on the downward-pointing arrow) and pseudogenization (under the downward-pointing arrow).

A further syntenic analysis of clade I genes in euteleostei (Fig. 3) revealed that the translocation of these genes was due to gene duplication and subsequent pseudogenization. Therefore, clade I genes were translocated in the same manner as that in retrocopy, namely via “copy & paste-style translocation”.

We found that some euteleostean species had clade I genes (*HCEs*) at another genomic location, in addition to the original genomic location in euteleostei, and described four patterns of genomic synteny as follows: (1) In salmon, *HCEs* were translocated into the conserved region in which *anxa2a*, *roraa*, and *otud7a* were arranged in this order (Fig. S6A). (2) In tilapia and cichlid, *HCEs* were translocated into the conserved region containing *glrx5*, *prkrip1*, and *serpina1l* arranged in this order (Fig. S6B). (3) In croaker, seabass, and stickleback, *HCEs* were translocated into the conserved region featuring *adgra1a*, *acbd4*, and *llgl2* in this order (Fig. S6C). (4) Finally, in *Takifugu* and *Tetraodon*, *HCEs* were translocated into the conserved region containing *fer1l4*, *cpne1*, and *nfs1* in this order (Fig. S6D). The positions of *HCEs* after such a translocation event were commonly found to be the same among closely related species, but not among distantly related ones. These results indicated that clade I genes (*HCEs*) further translocated via their ancestral position (light blue square in Fig. 3A) to each different position (yellow, green, purple, and brown squares in Fig. 3A), and these events independently occurred at least four times during euteleostei evolution.

We next compared the genomic location and the degree of vestiges of *HCEs* among species (Fig. 3B). There were three patterns of location and sequence of *HCEs* as follows: (i) Species that retain full-length *HCEs* at both the ancestral (parent position) and new (daughter position) locations. (ii) Species that retain full-length *HCE* at the daughter position, while retaining its vestiges (or even disappeared vestiges) at the parent position. (iii) Species that retain *HCE* only at the parent position. In croaker [a case (i) species], *HCE* sequences at each position showed a high similarity (90% amino acid homology); therefore, it seemed that *HCEs* at the daughter position were caused by the gene duplication of *HCEs* at the parent position (red balloon in Fig. 3B). In addition, in seabass and stickleback [case (ii) species], species related to croaker, which retained *HCEs* at the same daughter position, *HCEs* at the parent position were fragmented. Furthermore, in tilapia, *HCE* became shorter than that in seabass and stickleback, whereas no fragment was found in cichlid. Because traces of gene duplication and subsequent pseudogenization were found in some lineages, it seemed that the translocation of clade I genes was the cause of them (Fig. 3C).

### Genome synteny of clade II (LCE) genes

In addition to the results obtained by analyzing clade I, the findings for clade II also supported the hypothesis that intron loss of *HE* genes was caused by retrocopy (Fig. S7). The genomic synteny around the *LCEs* that were acquired in the common ancestor of the euteleostei was highly conserved, also including vestiges in ovoviviparous fishes, platyfish, and molly (Fig. S7A). In salmon, although the orders of neighboring genes in the genome were highly conserved, *LCEs* were not found at corresponding regions (Fig. S7A). Salmon *LCEs* were located on different chromosomes, but no *LCEs* were found at this location in other species, although the order of neighboring genes was highly conserved (Fig. S7B). As summarized in Fig. S10, after the acquisition of *LCE* (blue arrow), it seems that *LCE* was translocated and lost its introns (blue arrowhead) in the ancestor of salmon.

The synteny analysis performed in this study showed the relationship between the translocation and intron loss of *HEs*, and revealed that this relationship was causative, namely the intron loss occurred in a retrocopy-dependent manner. However, it remains unclear how the expression of these genes was maintained. We next focused on the promoters of clade I genes, which are conserved among all oviparous teleosts and were translocated several times.

### Promoter assay of clade I

**Figure 4:**
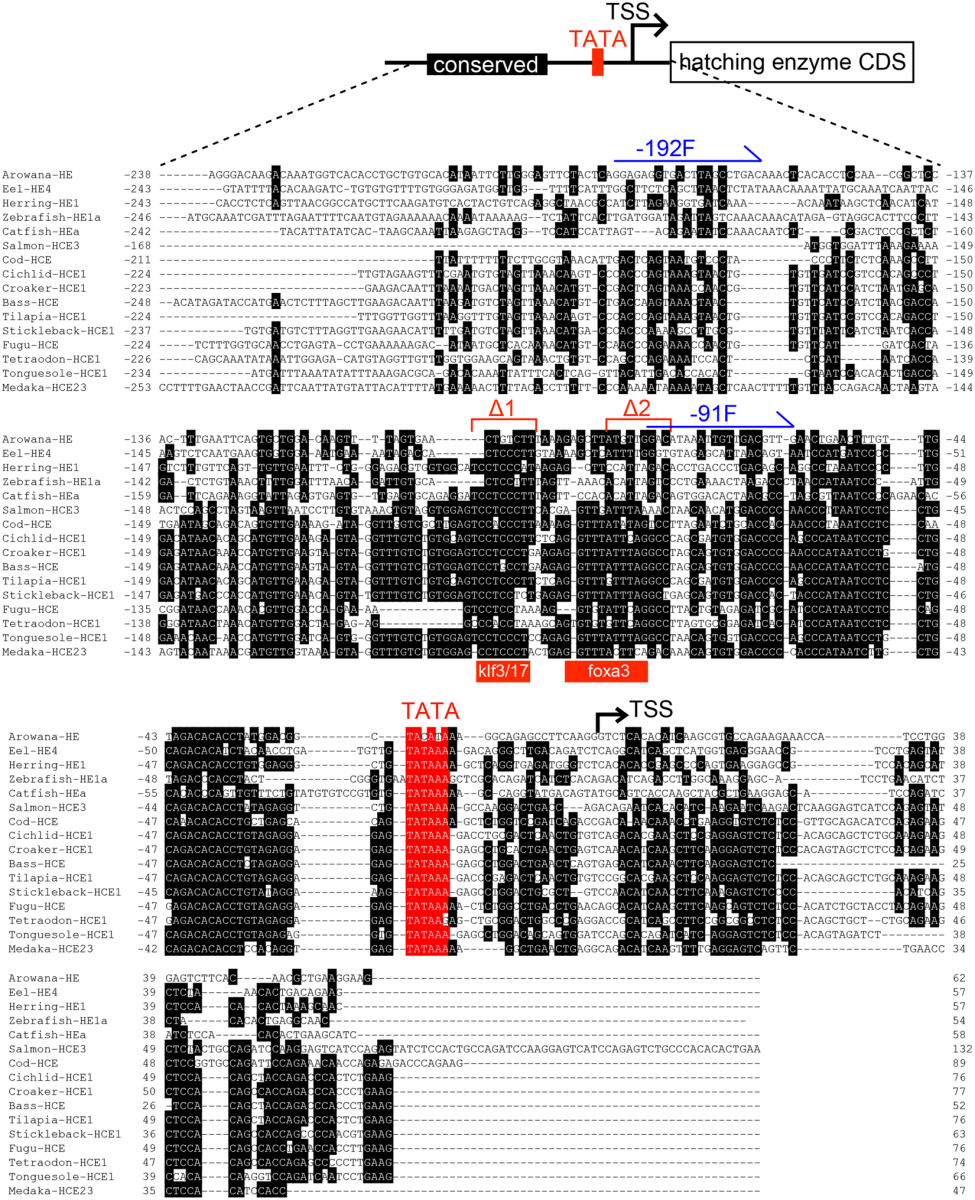
Conserved upstream sequences of clade I genes. The upper part presents a schematic drawing of the coding sequence (CDS) and the upstream sequence. The lower part presents the alignment of the upstream sequences of clade I genes. Transcription start site (TSS), putative binding sites of transcription factors, TATA box (TATA), and conserved sequences are indicated as a folding arrow, red bars, red square, and black squares, respectively. The position of TSS is indicated with reference to zebrafish.

**Figure 5:**
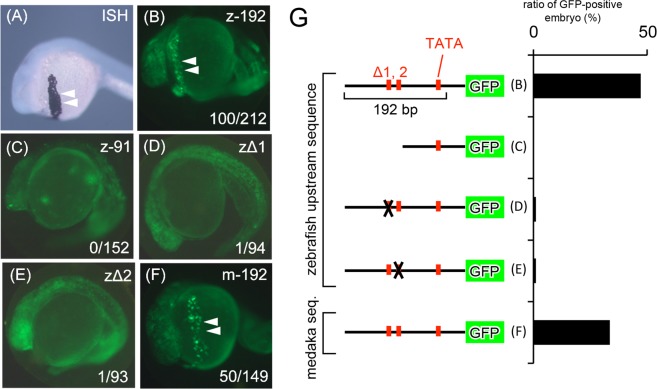
Reporter assay of clade I genes (**A**) *in situ* hybridization (ISH) of *HE* in pre-hatching zebrafish. (**B**) Pre-hatching zebrafish injected with the GFP construct containing the conserved upstream sequence of zebrafish (z-192). (**C**) Pre-hatching zebrafish injected with the GFP construct lacking the conserved upstream sequence of zebrafish (z-91). Zebrafish injected with the GFP construct lacking the putative binding site of klf3/17 (**D**) and foxa3. (**E**,**F**) Pre-hatching zebrafish injected with the GFP construct containing the conserved upstream sequence of medaka (m-192). The length of the upstream sequence used for the analysis indicates the distance from the TSS of zebrafish and medaka. Arrowheads indicate positive signals in hatching gland cells. The numbers at the bottom right in b–f indicate the ratio of the embryo displaying positive signals (positive/injected). (**G**) Summary of results and constructs using this reporter assay.

Interestingly, although the clade I genes were translocated to other locations, the sequences upstream of them were highly conserved in each species (Fig. 4), and these conserved sequences displayed promoter activity (Fig. 5). The 16 teleosts had conserved sequences of approximately 200 bp, including a TATA box, upstream of the transcription start site (TSS; Fig. 4). These sequences contained some putative binding sequences of the transcription factors expressed in hatching gland cells (red bars in Fig. 4), including *klf3*^29^, *klf17*^30^, and *foxa3*^31^ [the putative binding site of *klf3/17* is CACCC or CTCCC^32^, whereas that of *foxa3* is TGTTT(A/G)C(T/A)(T/C)(A/T)]^33^. Moreover, phylogenetic analysis using these conserved sequences produced findings that were roughly consistent with the evolutionary relationships already revealed in teleosts (Fig. S8). These results indicate that, when *HEs* were translocated, the upstream region of the TSS accompanied them.

To investigate the role of these conserved sequences in gene expression, we connected the upstream sequence of the zebrafish to GFP as a reporter, and microinjected this construct into zebrafish eggs (Fig. 5). As reported previously^34^, *hatching enzyme genes* (*he1*) are expressed in hatching gland cells, which are cells specialized in the synthesis and secretion of *HEs* located at the surface of the egg yolk (Fig. 5A). As shown below, we next conducted the reporter assay by using some constructs in which the GFP was linked to the upstream sequence of zebrafish *HE* and medaka *HCE* (shown in Fig. 5B–F, and summarized in Fig. 5G). The zebrafish into which the conserved upstream sequences (−192) had been injected displayed GFP-positive signals in hatching gland cells (Fig. 5B), whereas the shorter sequences (−91), lacking putative transcription factor binding sites, were not associated with any such signal (Fig. 5C). These results suggest that the 200-bp upstream region (or more specifically, the region of −192 to −91) is indispensable for *HE* expression. Next, we prepared constructs lacking the putative binding site for transcription factors expressed in hatching gland cells (Δ1 and Δ2 in Fig. 4). No GFP signal was observed in any of the analyses using these constructs (Fig. 5D,E). A previous study showed that zebrafish with *klf17* knockdown displayed a drastic decline of the expression of *HEs*^30^, which is consistent with our results obtained using the Δ2 construct. Furthermore, the expression of *foxa3* in hatching gland cells occurs downstream of *klf17*^35^; therefore, it is expected that *foxa3* may also be involved in regulating *HE* expression. Thus, these conserved upstream sequences are considered to be promoter sequences of *HE*. Finally, we used the upstream sequence of medaka *HCE*, which shared such indispensable sequences (putative binding site of *klf3/17* and *foxa3*) although they are located at a different location from those in zebrafish and have lost all of their introns. The zebrafish embryo injected with the medaka construct exhibited GFP signals in hatching gland cells (Fig. 5F), which indicated that when *HEs* were translocated, they were accompanied by the promoter sequences.

## Discussion

The precise regulation of the correct expression of genes at numerous coding regions across the genome is one of the most important factors for life. Considering that the regulation of gene expression is affected by promoters and chromatin structure, it was conventionally thought that the maintenance of a consistent position in the genome is key to ensure consistency in gene regulation over the course of evolution^36,37^. For example, the *Hox* gene clusters, which are an important set of genes for somitogenesis, and some noncoding sequences (including promoters and enhancer) have maintained their location in the genome (including their neighboring genes) during the evolution of vertebrates^38,39^. Therefore, the phenomenon that *HEs* have maintained their system of expressional regulation while frequently changing their genomic location is surprising.

**Figure 6:**
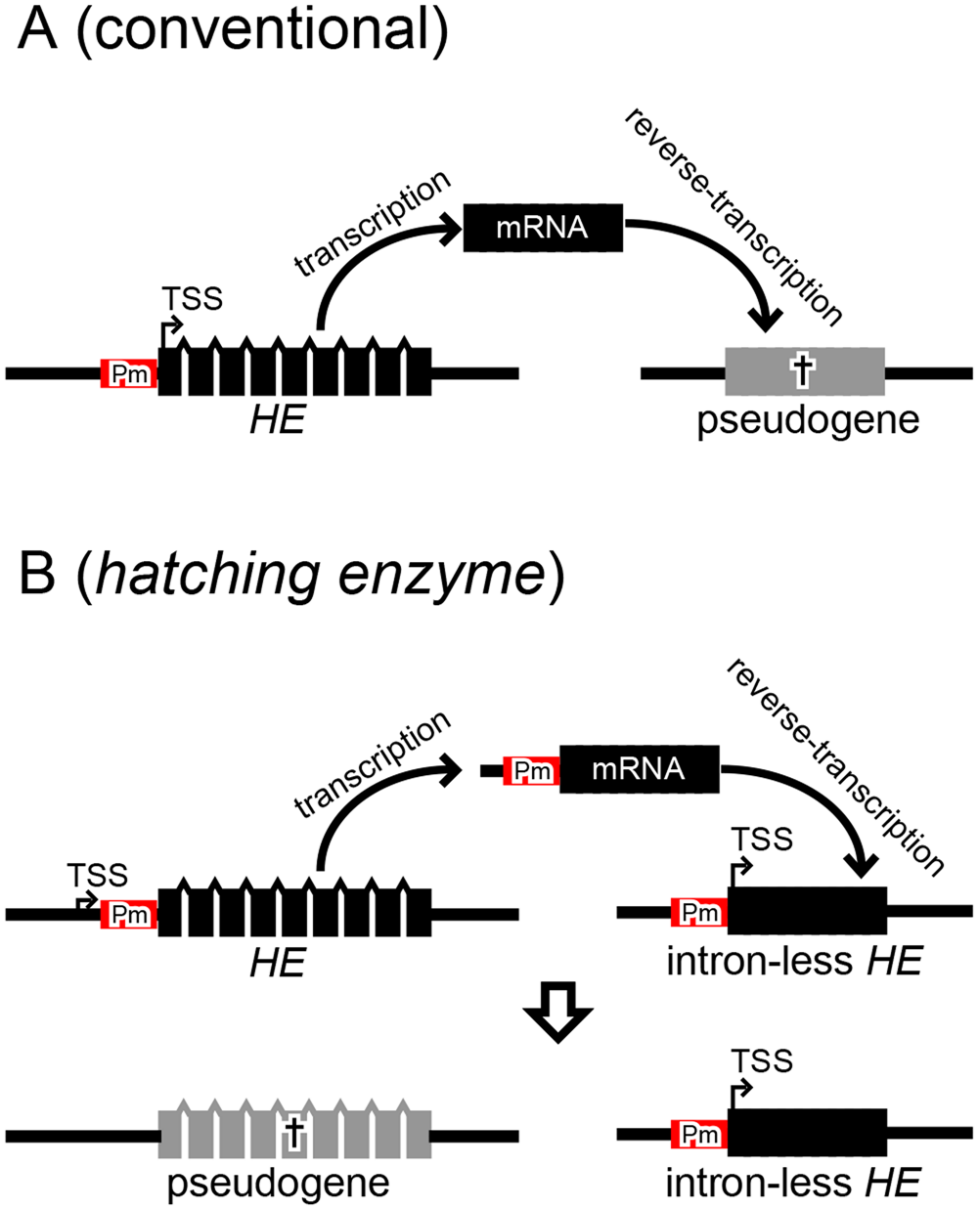
The hypothetical model of the evolution of intron-loss by retrocopy in teleostean *HEs*. (**A**) Conventional retrocopying. The retrocopied daughter genes generally lose the promoter and become pseudogenes. (**B**) Retrocopying of *HEs* in teleostei. *HEs* were retrotranslocated with the promoter sequence because additional TSS was obtained upstream of the promoter. Pm indicates the original promoter of *HEs*.

In addition to the above phenomenon, our results also shed light on the mechanism behind another unique feature, namely the loss of introns of *HEs* accompanied by the maintenance of their promoters. We here propose a model explaining the mechanism behind this (Fig. 6). Specifically, in conventional retrocopy, retrocopied genes lose their promoter and become pseudogenes, whereas *HEs* became active intron-less genes by retrocopying abnormal transcripts containing the promoter sequence. In support of this, from their analysis involving a comparison of genomic sequences, Okamura and Nakai also reported that some active retrocopied genes in humans have CpG islands, which usually display weak promoter activity; thus, they proposed the presence of a new retrocopy system that retains the original promoter during retrocopy^21^. Our results from the promoter assay confirmed that an active promoter is maintained after retrocopy, and strongly supported the above hypothesis. In recent years, a report was published describing that anchovies, which are related to herring and have intron-containing *HEs*, express some splicing variants of *HEs* in the ovary^40^. Although there is no detailed mention of TSS in this report, this implies that the *HE* containing promoter encompasses the potential to express in the ovary. In the present study, we also detected the spliced mRNA in ovarian cDNA of zebrafish, which contained partial promoter sequences of TSS (Fig. S9). Although this finding requires further examination, it may presently provide us with a clue for how retrocopied promoter containing spliced mRNA may be inherited.

Our results suggest that *HEs*, even after duplication, can easily retrotranslocate and transform into intron-less genes, although it is unclear why retrocopy preferentially occurs for *HEs*. Generally, the reverse transcriptase of retrotransposons recognizes the “stringent recognition sequence” at the 3′ end, to initiate reverse transcription^41^. In mammals, the recognition of the reverse transcriptase of LINE-1, a kind of autonomous retrotransposon, exceptionally became loose (poly-A tail target); therefore, the frequency of the retrocopy was drastically increased^42^. As a result, in mammals, considerable numbers of processed pseudogenes are found in the genome, whereas in teleosts, it has been thought that intron-less genes are rare because of the low frequency of retrocopy^43^. To investigate why teleostean *HEs* are a preferential target for reverse transcriptase, we searched for its recognition site on *HEs*, but failed to find a sequence (trace) similar to the reverse transcriptase recognition site at the 3′-end recognition site of *HEs*, potentially because of the accumulation of mutations during the long evolutionary period or a disturbance by tandem duplication. To clarify why *HEs* were able to retrotranslocate so frequently, there is a need to perform more comparisons of this issue among related species.

Finally, we discuss the effects of retrocopying on the molecular evolution of *HEs* and on reproduction. The egg envelope, which is in direct contact with the external environment, has various thicknesses, sizes, and levels of hardness depending on fish species, and it is believed that this egg envelope diversity enables successful reproduction in various environments^44–46^. For obtaining this divergence of the egg envelope, rapid evolution of the egg envelope protein, which is the main component of the egg envelope, and co-evolution of *HE*, in a clearly one-to-one relationship with the egg envelope as an enzyme and substrate, are indispensable^16,47,48^. However, it is well known that the molecular evolution of functionally important genes is generally slow^49^. Also for this molecular co-evolution, it is necessary for both a mutation around the cleavage site of the egg envelope protein and a mutation that changes the substrate specificity of HE to occur. Upon the failure of such co-evolution to occur, the activity of HEs to digest the egg envelope is lost, which means that reproduction cannot occur. Thus, there are certain restrictions that hinder molecular co-evolution of HE and egg envelope protein. However, several studies have reported rapid molecular co-evolution of HE and egg envelope protein^16,47^. In particular, HEs from one species do not act (or show low activity) on the egg envelope of another, so it has been strongly considered that the specificity among HEs and egg envelope proteins changed^34,50,51^. Accordingly, it is expected that frequent retrocopy might have promoted the molecular evolution of *HEs*. One of the most influential forces behind the evolution of such important genes is gene duplication^52^. Retrocopying is a type of gene duplication mediated by mRNA, and has the ability to insert multiple copies of genes at multiple sites in the genome^53,54^. It might have been that frequent retrocopy of *HEs* raised the molecular evolution rate of *HEs*, allowed variety of teleostean egg envelope protein, and allowed teleost reproduction in various environments.

## Methods

### Ethical statement

All animal experiments were approved by the Institutional Animal Care and Experimentation Committee (IACUC, approval identification number 2016-081) at The Jikei University School of Medicine, compiled with the Guide for the Care and Use of Laboratory Animals of the IACUC. All experiments in this study was performed in accordance with the relevant guidelines and regulations.

### Animals

The fertilized eggs that were naturally spawned were collected from adult zebrafish (*Danio rerio*) and medaka (*Oryzias latipes*), which were reared at 25 °C. To collect genomic DNA and total RNA from ovaries, adult zebrafish and medaka were sacrificed under anesthesia induced by tricaine (MS-222). Japanese eels (*Anguilla japonica*) obtained from a commercial supplier were also used for the extraction of genomic DNA.

### *In silico* cloning and comparison of genomic synteny

The sequence data of the teleostean genome registered in Ensembl (www.ensembl.org/)^55^ were used for comparative genomic analysis. In addition to these data, the genomic data of Asian arowana (*Scleropages formosus*)^56^, Japanese eel (*Anguilla japonica*)^57^, Atlantic herring (*Clupea harengus*)^58^, channel catfish (*Ictalurus punctatus*)^59,60^, Atlantic salmon (*Salmo salar*)^61,62^, large yellow croaker (*Larimichthys crocea*)^63^, African cichlid (*Maylandia zebra*)^64^, European seabass (*Dicentrarchus labrax*)^65^, and half-smooth tonguesole (*Cynoglossus semilaevis*)^66^ registered in NCBI were also used. To determine the full-length coding sequences of the homologous genes, *in silico* cloning was conducted as follows. First, we selected the species for which the complete coding sequence of the gene of interest had already been obtained and for which the whole genome sequence had already been published. Second, the exon–intron structures of the genes of interest were determined by comparing the coding sequence and genomic sequence in accordance with the GT-AG rule^67^. Subsequently, the coding sequences of the other species were determined by aligning the exonic sequences of related species. To draw putative conclusions about the evolutionary transition of *HEs*, a comparison of the genomic synteny around *HEs* was performed among the species. The genomic synteny was basically compared with reference to Genomicus ver. 88.01^68,69^, but as some information was lacking, we obtained a greater volume of information for more accurate comparison. We estimated the neighboring genes from the information of other species, searched by BLASTX, and cloned *in silico* to obtain information on the genes overlooked in the prediction in Ensembl. In the species for which data of the genomic sequences were registered only in NCBI, the sequences of the neighboring genes were determined in the same way (combination of information from blastx and other species). In the eel genome, we amplified the sequences of the corresponding region by PCR from the genomic DNA using primers specific for the region between the predicted neighboring gene (*kcnk4*) and *HE*, and performed sequencing using DNA-sequencer 3130 (Applied Biosystems, CA, USA). The phylogenetic relationship of teleostean species was used for comparison of genomic synteny, in accordance with a previous study^1^.

### Phylogenetic analysis of HEs and their upstream sequences

The phylogenetic trees were basically structured in accordance with a slightly modified version of a method used in a previous study^13^. After alignment of the nucleotide sequences using Clustalx program ver. 2.0^70^, the trees were constructed using the maximum likelihood method in the program RAxML ver. 8^71^ with GTR + Γ + I as a model. To evaluate the reliability of the nodes of the trees, bootstrap values were calculated from 1000 repetitions. In the case of *HEs*, sequence alignments of the protease domain were realigned using the CodonAlign 2.0 program to separate the first, second, and third positions of each codon. A tree for the region upstream of *HEs* was constructed using the region approximately 100 bp upstream from the TATA box.

### Reporter assay of the promoter of HEs

We analyzed the promoter activity of the region upstream of *HEs* by using the GFP protein as a reporter. Amplified fragments from medaka and zebrafish genomic DNA were inserted into a GFP vector (pT2AL200R150G)^72^ provided by Prof. Koichi Kawakami of the National Institute of Genetics, Japan. These GFP constructs were microinjected with synthesized transposase mRNA into fertilized zebrafish eggs. The promoter activities were calculated on the basis of the presence or absence of GFP fluorescence in hatching gland cells of zebrafish embryos at 24–36 h after fertilization. To visualize the localization of hatching gland cells and use them for comparison, *in situ* hybridization of *HEs* was performed, in accordance with our previously described method^8^.

### Detection of long-chain transcripts of HEs in ovary

To detect the transcripts containing the upstream region of the original TSS, RT-PCR was conducted using ovarian RNA. In zebrafish, to determine whether the PCR product was derived from mRNA or genomic DNA, the DNA primers were designed to be specific for positions either side of the intron to amplify fragments of different sizes depending on their origin. Because we could not distinguish medaka *HCE* by size because it lacks introns, we also used DNase treatments of total RNA. These treatments were performed in accordance with the attached manual (Invitrogen, MA, USA).

## Supplementary information

Supplementary Information
