# Supplementary material for: Translocation of promoter-conserved hatching enzyme genes with intron-loss provides a new insight in the role of retrocopy during teleostean evolution
Source: Sci Rep. 2019 Feb 21;9:2448. doi: 10.1038/s41598-019-38693-6 (PMC6385490; doi:10.1038/s41598-019-38693-6)
Supplement: Supplementary file 1 — Supplementary Information [file 41598_2019_38693_MOESM1_ESM.pdf]

Supplementary information

Supplementary figures (Fig. S1–S9 and Table S1)

Title

Translocation of promoter-conserved hatching enzyme genes with intron-loss provides a new insight in the role of retrocopy during teleostean evolution

Authors

Tatsuki Nagasawa, Mari Kawaguchi, Tohru Yano, Sho Isoyama, Shigeki Yasumasu*, Masataka Okabe

Detection of the long transcripts of HEs

RT-PCR showed that the ovary (germ organ) of the zebrafish expressed long transcripts, which contained sequences that are upstream of normal TSS (Fig. S9). We conducted RT-PCR by using ovarian RNA of medaka and zebrafish. Upstream and downstream primers were designed from the sequences of the promoter regions and 3′-coding regions, respectively (Fig. S9A and B). No band was detected after 30 cycles of PCR (not shown). However, after 35 cycles, clear bands were amplified from the RNA of both ovaries (without DNase treatment in Fig. S9C and D). In zebrafish, two bands of different sizes derived from intron-containing genomic DNA (upper bands) and spliced mRNA (lower bands) were detected (Fig. S9C). DNase treatment of ovarian RNA followed by PCR resulted in diminution of the upper bands and enhancement of the lower ones (Fig. S9C). In medaka, the amplified bands were diminished by DNase treatment of RNA (Fig. S9D). These results indicate that the long transcripts containing a partial promoter sequences were weakly but definitely expressed in zebrafish ovary.

Fig. S1 Multiple alignment of newly cloned hatching enzymes

The primary sequences of the hatching enzymes for herring (*Clupea herengus*), catfish (*Ictalurus panctatsu*), cichlid (*Maylandia zebra*), croaker (*Larimichthys crocea*), seabass (*Dicentrarchus labrex*), and tonguesole (*Cynoglossus semilaevis*) were aligned by using clustalW program. Identical residues, more than 50%, are boxed. Each sequences contained consensus sequences, conservatively found in hatching enzymes, are highlighted with gray (active sites) and black (cysteine).

Fig. S2 Phylogenetic tree of *HEs* in teleostean species

A maximum likelihood phylogenetic tree was constructed using nucleotide sequences of the protease domain of *HEs* after realignment based on the position of the codon. Each species is described using the common name; those newly cloned in this study are highlighted with red letters. The accession numbers of sequences used to construct this tree are listed in Table S1. The branch length, defined as per the scale bar, represents the mutation rate. The number at each node indicates the bootstrap value.

Fig. S2 Alignments of the vestiges of *HCEs* (clade I genes) and LCE (clade II genes) in molly and medaka

Two sequences of vestiges of *HCEs* (A and B) and *LCE* (C) in molly were aligned with medaka *HCE* and *LCE*, respectively. Small letters indicate nucleotide sequences of vestiges of hatching enzymes, and capital letters above them are three reading frames of amino acids corresponding to the nucleotides. Stop codons are indicated as asterisks. The amino acid sequences of medaka hatching enzymes, above the three reading frames, are boxed and aligned to the three reading frames. Reading frames corresponding to medaka hatching enzymes are boxed, and matching amino acids and stop codons are highlighted as black and red, respectively. Because *LCE* retains its introns, unlike intron-less *HCE*, the exon numbers are indicated with red circles.

Fig. S4 Alignments of the vestiges of *HCE* (clade I genes) in perciformes and own full-length *HCE*

Sequences of vestiges of *HCE* of (**A**) seabass, (**B**) stickleback, and (**C**) tilapia are aligned with their own full-length HCEs as in Figure S3.

Fig. S5 Genome syntenies around clade I hatching enzymes

Genome syntenies around clade I hatching enzymes in (A) arowana and herring, (B) otophysi, and (C) ancestral location of clade I *HCEs* in eutelostei were schematically drawn. Triangles indicate the coding region of genes. Red triangles indicate the coding region of *HEs*. The direction in which the tip of the triangle is pointing represents the direction of transcription. The size of genes and

the distance between genes are relatively indicated on the basis of the scale bar. Gray triangles with a cross indicate vestiges of *HCEs*, and scales of these vestiges are not considered. The species' common names are listed on the left, and the corresponding numbers of chromosomes, scaffolds, or linkage groups are listed with them.

Fig. S6 Genome synteny around the lineage-specific locations of clade I *HCEs* in euteleostei

Genome synteny around the lineage-specific location of clade I *HCEs* in (**A**) salmon; (**B**) tilapia and cichlid; (**C**) croaker, seabass, and stickleback; and (**D**) *Tetraodon* and fugu is schematically drawn as in Fig. S5.

Fig. S7 Genome synteny around LCE

Genome synteny around LCEs in (**A**) euteleostei except salmon, and in (**B**) salmon, is schematically drawn as in Fig. S5, replacing red with blue. (**C**) Evolutionary scheme of the genomic location of clade II (*LCE*) in teleostei. The orders of genes around *LCEs* (in Fig. S7A and B) are summarized as in Figure 1, replacing red with blue. The evolutionary timing of the acquisition of *LCE* is indicated by an arrow. Two corresponding chromosomes are lined up only in tetraploid salmon.

Fig. S8 Phylogenetic tree produced using upstream sequences of TSS of clade I genes

A maximum likelihood tree was constructed using a nucleotide sequence approximately 100-bp upstream of the TATA box. The branch length, defined by the scale bar, represents the mutation rate. The number at each node indicates the bootstrap value.

Fig. S9 Detection of long transcripts in ovarian RNA

Primer maps to detect transcripts containing the upstream region in ovary of (**A**) zebrafish and (**B**) medaka. Squares, solid line, and broken line indicate the exonic, intronic, and flanking regions of *HEs*. *HEs* are normally transcribed from the transcription start site (TSS). Detection of transcripts containing the upstream region in (**C**) zebrafish and (**D**) medaka ovarian RNA by RT-PCR. DNA ladder marker bands indicate 2000, 1650, 1000, 850, 600, 500, 400, 300, 200, and 100 bp. Arrows on the right side indicate amplification products from DNA and RNA.

Table S1 The accession numbers of hatching enzyme genes from Fig. S2.

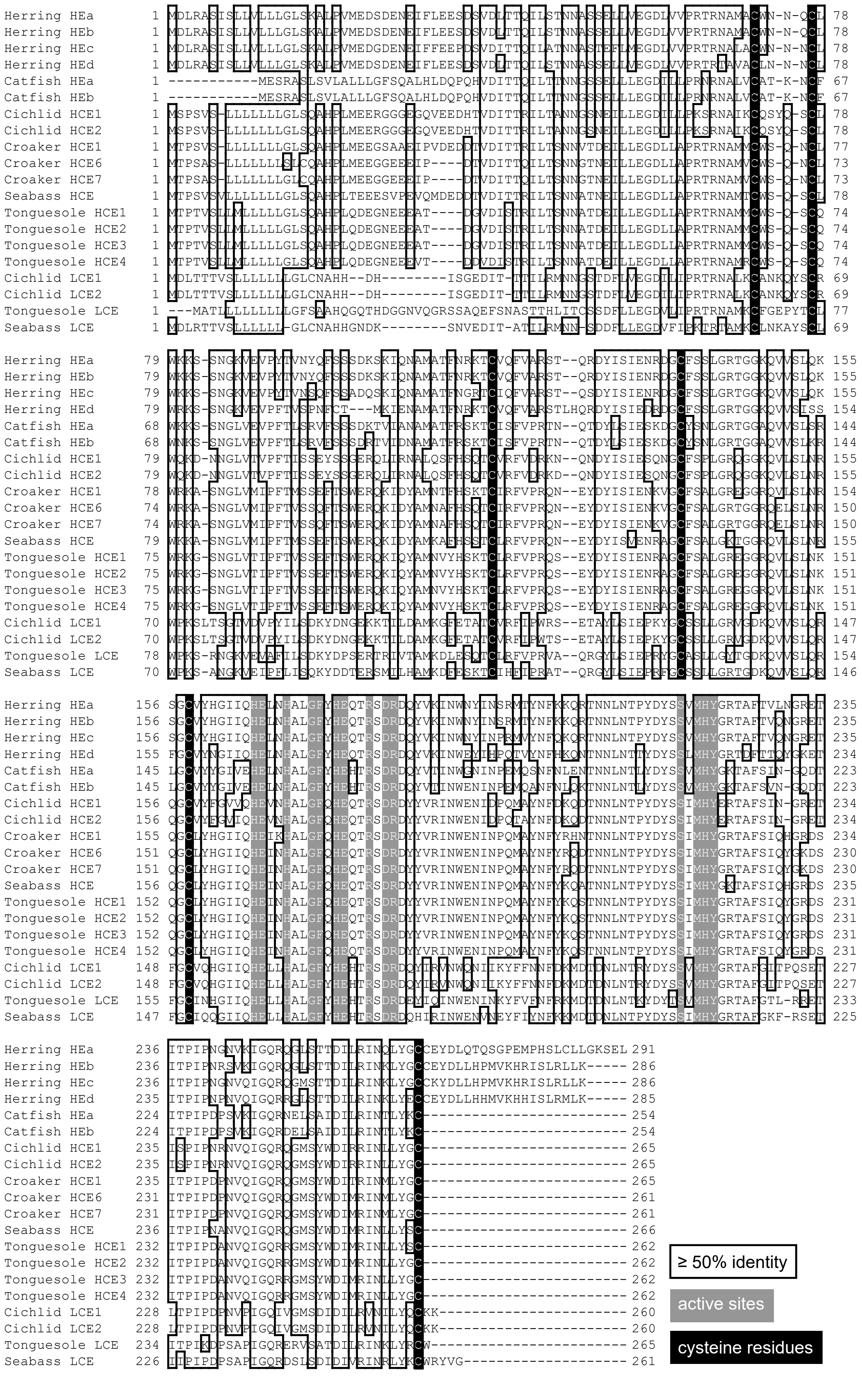
Herring HEa 1 MDLRASISLLVLLLGLSKALPVMEDSDENEIFLEESDSVDLTTQILSTNNASSELLVEGDLVVPRTRNAMACWN-N-QCL 78
Herring HEb 1 MDLRASISLLVLLLGLSKALPVMEDSDENEIFLEESDSVDLTTQILSTNNASSELLVEGDLVVPRTRNAMACWN-N-QCL 78
Herring HEc 1 MDLRASISLLVLLLGLSKALPVMEDGDENEIFFEEPDSVDITTQILATNNASTEFLMEGDLVVPRTRNALACWN-N-NCL 78
Herring HEd 1 MDLRASISLLVLLLGLSKALPVMEDSDENEIFLEESDSVDLTTQILSTNNASSELLVEGDLVVPRTRTAVACLN-N-NCL 78
Catfish HEa 1 -----------MESRASLSVLALLLGFSQALHLDQPQHVDITTQILTTNNGSSELLLEGDILLPRNRNALVCAT-K-NCF 67
Catfish HEb 1 -----------MESRASLSVLALLLGFSQALHLDQPQHVDITTQILTTNNGSSELLLEGDILLPRNRNALVCAT-K-NCF 67
Cichlid HCE1 1 MSPSVS-LLLLLLLGLSQAHPLMEERGGGEGQVEEDHTVDITTRILTANNGSNEILLEGDILLPKSRNAIKCQSYQ-SCL 78
Cichlid HCE2 1 MSPSVS-LLLLLLLGLSQAHPLMEERGGGEGQVEEDHTVDITTRILTANNGSNEILLEGDILLPKSRNAIKCQSYQ-SCL 78
Croaker HCE1 1 MTPSVS-LLLLLLLGLSQAHPLMEEGSAAEIPVDEDDTVDITTRILTSNNVTDEILLEGDLLAPRTRNAMMCWS-Q-NCL 77
Croaker HCE6 1 MTPSAS-LLLLLLLSLCQAHPLMEEGGEEEIP----DTVDITTQILTSNNGTNEILLEGDLLAPRTRNAMVCWS-Q-NCL 73
Croaker HCE7 1 MTPSAS-LLLLLLLGLCQAHPLMEEGGEEEIP----DTVDITTQILTSNNGTNEILLEGDLLAPRTRNAMVCWS-Q-NCL 73
Seabass HCE 1 MTPSVSVLLLLLLLGLSQAHPLTEEESVPEVQMDEDDTVDITTRILTSNNATDEILLEGDLLAPRTRNAMICWS-Q-SCL 78
Tonguesole HCE1 1 MTPTVSLLMLLLLGLSQAHPLQDEGNEEEAT----DGVDISTRILTSNNATDEILLEGDLLAPRTRNAMMCWS-Q-SCQ 74
Tonguesole HCE2 1 MTPTVSLLMLLLLGLSQAHPLQDEGNEEEAT----DGVDISTRILTSNNATDEILLEGDLLAPRTRNAMMCWS-Q-SCQ 74
Tonguesole HCE3 1 MTPTVSLLMLLLLGLSQAHPLQDEGNEEEAT----DGVDISTRILTSNNATDEILLEGDLLAPRTRNAMMCWS-Q-SCQ 74
Tonguesole HCE4 1 MTPTVSLLMLLLLGLSQAHPLQDEGNEEEVT----DDVDISTRILTSNNATDEILLEGDLLAPRTRNAMRCWS-Q-SCQ 74
Cichlid LCE1 1 MDLTTTVSLLLLLLLGLCNAHH--DH--------ISGEDIT-TTILRMNNGSTDFLVEGDILIPRTRNALKCANKQYSCR 69
Cichlid LCE2 1 MDLTTTVSLLLLLLLGLCNAHH--DH--------ISGEDIT-TTILRMNNGSTDFLVEGDILIPRTRNALKCANKQYSCR 69
Tonguesole LCE 1 ---MATLLLLLLLLGFSAAHQGQTHDGGNVQGRSSAQEFSNASTTHLITCSSDFLLEGDVLIPRTRNAMKCFGEPYTCL 77
Seabass LCE 1 MDLRTTVSLLLLLL-GLCNAHHGNDK--------SNVEDIT-ATILRMNN-SDDFLLEGDVFIPKTRTAMKCLNKAYSCL 69
Herring HEa 79 WKKS-SNGKVEVPYTVNYQFSSSDKSKIQNAMATFNRKTCVQFVARST--QRDYISIENRDGCFSSLGRTGGKQVVSLQK 155
Herring HEb 79 WKKS-SNGKVEVPYTVNYQFSSSDKSKIQNAMATFNRKTCVQFVARST--QRDYISIENRDGCFSSLGRTGGKQVVSLQK 155
Herring HEc 79 WKKS-SNGKVEVPYTVNSQFSSADQSKIQNAMATFNGRTCIQFVARST--QSDYISIENRDGCFSSLGRTGGKQVVSLQK 155
Herring HEd 79 WRKS-SNGKVEVPFTVSPNFCT---MKIENAMATFNRKTCVQFVARSTLHQRDYISIEDRDGCFSSLGRTGGKQVVSISS 154
Catfish HEa 68 WKKS-SNGLVEVPFTLSRVFSSSDKTVIANAMATFRSKTCISFVPRTN--QTDYLSIESKDGCYSNLGRTGGAQVVSLSR 144
Catfish HEb 68 WNKS-SNGLVEVPFTLSRVFSSSDRTVIDNAMATFRSKTCISFVPRTN--QTDYLSIESKDGCYSNLGRTGGAQVVSLKR 144
Cichlid HCE1 79 WQKD-NNGLVTVPFTISSEYSSGERQLIRNALQSFHSQTCVRFVDRKN--QNDYISIESQNGCFSPLGRQGGKQVLSLNR 155
Cichlid HCE2 79 WQKD-NNGLVTVPFTISSEYSSGERQLIRNALQSFHSQTCVRFVDRKD--QNDYISIESQNGCFSPLGRQGGKQVLSLNR 155
Croaker HCE1 78 WRKA-SNGLVMIPFTMSSEFTSWERQKIDYAMNTFHSKTCIRFVPRQN--EYDYISIENKVGCFSALGREGGRQVLSLNR 154
Croaker HCE6 74 WRKA-SNGLVMIPFTVSSQFTSWERQKIDYAMNAFHSQTCIRFVPRQN--EYDYISIENKVGCFSALGRTGGRQELSLNR 150
Croaker HCE7 74 WRKA-SNGLVMIPFTVSSQFTSWERQKIDYAMNAFHSQTCIRFVPRQN--EYDYISIENKVGCFSALGRTGGRQELSLNR 150
Seabass HCE 79 WKKA-SNGLVMIPFTMSSEFTSWERQKIDYAMKAFHSKTCIRFVPRQN--EYDYISVENRAGCFSALGKTGGRQVLSLNR 155
Tonguesole HCE1 75 WRKG-SNGLVTIPFTVSSEFTSWERQKIQYAMNVYHSKTCLRFVPRQS--EYDYISIENRAGCFSALGREGGRQVLSLNK 151
Tonguesole HCE2 75 WRKG-SNGLVTIPFTVSSEFTSWERQKIQYAMNVYHSKTCLRFVPRQS--EYDYISIENRAGCFSALGREGGRQVLSLNK 151
Tonguesole HCE3 75 WRKG-SNGLVTIPFTVSSEFTSWERQKIQYAMNVYHSKTCLRFVPRQS--EYDYISIENRAGCFSALGREGGRQVLSLNK 151
Tonguesole HCE4 75 WRKG-SNGLVTIPFTVSSEFTSWERQKIQYAMNVYHSKTCLRFVPRQS--EYDYISIENRAGCFSALGREGGRQVLSLNK 151
Cichlid LCE1 70 WPKSLTSGIVDVPYILSDKYDNGEKKTILDAMKGFETATCVRFIPWRS--ETAYLSIEPKYGCSSLLGRVGDKQVVSLQR 147
Cichlid LCE2 70 WPKSLTSGIVDVPYILSDKYDNGEKKTILDAMKGFETATCVRFIPWTS--ETAYLSIEPKYGCSSLLGRVGDKQVVSLQR 147
Tonguesole LCE 78 WPKS-RNGKVEVAFILSDKYDPSERTRIVTAMKDLESQTCLRFVPRVA--QRGYLSIEPRYGCASLLGYTGDKQVVSLQR 154
Seabass LCE 70 WPKS-ANGKVEIPFLISQKYDDTERSMILHAMKDFESKTCIHFTPRAT--QRGYLSIEPRFGCSSLLGRTGDKQVVSLQR 146
Herring HEa 156 SGCVYHGIIQHELNHALGFYHEQTRSDRDQYVKINWNYINSRMTYNFKKQRTNNLNTPYDYSSVMHYGRTAFTVLNGRET 235
Herring HEb 156 SGCVYHGIIQHELNHALGFYHEQTRSDRDQYVKINWNYINSRMTYNFKKQRTNNLNTPYDYSSVMHYGRTAFTVQNGRET 235
Herring HEc 156 SGCVYHGIIQHELNHALGFYHEQTRSDRDQYVKINWNYINPRMVYNFQKQRTNNLNTPYDYSSVMHYGRTAFTVQNGRET 235
Herring HEd 155 FGCVYNGIIQHELNHALGFYHEQTRSDRDQYVKINWEYIHPQTVYNFHKQNTNNLNTTYDYSSLMHYGRTDFTTQYGKET 234
Catfish HEa 145 LGCVYYGIVEHELNHALGFYHEHTRSDRDQYVTINWGNINPEMQSNFNLENTNNLNTLYDYSSVMHYGKTAFSIN-GQDT 223
Catfish HEb 145 LGCVYYGIVEHELNHALGFYHEHTRSDRDQYVTINWENINPEMQANFNLQKTNNLNTLYDYSSVMHYGKTAFSVN-GQDT 223
Cichlid HCE1 156 QGCVYFGVVQHEVNHALGFQHEQTRSDRDYYVRINWENIDPQMAYNFDKQDTNNLNTPYDYSSIMHYERTAFSIN-GRET 234
Cichlid HCE2 156 QGCVYFGVIQHEVNHALGFQHEQTRSDRDYYVRINWENIDPQTAYNFDKQDTNNLNTPYDYSSIMHYERTAFSIN-GRET 234
Croaker HCE1 155 QGCLYHGIIQHEIKHALGFQHEQTRSDRDYYVRINWENINPQMAYNFYRHNTNNLNTPYDYSSIMHYGRTAFSIQHGRDS 234
Croaker HCE6 151 QGCLYHGIIQHEINHALGFQHEQTRSDRDYYVRINWENINPQMAYNFYRQDTNNLNTPYDYSSIMHYGRTAFSIQYGKDS 230
Croaker HCE7 151 QGCLYHGIIQHEINHALGFQHEQTRSDRDYYVRINWENINPQMAYNFYRQDTNNLNTPYDYSSIMHYGRTAFSIQYGKDS 230
Seabass HCE 156 QGCLYHGIIQHEINHALGFQHEQTRSDRDYYVRINWENINPQMAYNFYKQATNNLNTPYDYSSIMHYGKTAFSIQHGRDS 235
Tonguesole HCE1 152 QGCLYHGIIQHEINHALGFQHEQTRSDRDYYVRINWENINPQMAYNFYKQSTNNLNTPYDYSSIMHYGRTAFSIQYGRDS 231
Tonguesole HCE2 152 QGCLYHGIIQHEINHALGFQHEQTRSDRDYYVRINWENINPQMAYNFYKQSTNNLNTPYDYSSIMHYGRTAFSIQYGRDS 231
Tonguesole HCE3 152 QGCLYHGIIQHEINHALGFQHEQTRSDRDYYVRINWENINPQMAYNFYKQSTNNLNTPYDYSSIMHYGRTAFSIQYGRDS 231
Tonguesole HCE4 152 QGCLYHGIIQHEINHALGFQHEQTRSDRDYYVRINWENINPQMAYNFYKQSTNNLNTPYDYSSIMHYGRTAFSIQYGRDS 231
Cichlid LCE1 148 FGCVQHGIIQHELLHALGFYHEHTRSDRDQYIRVNWQNIIKYFFNNFDKMDTDNLNTRYDYSSVMHYGRTAFGITPQSET 227
Cichlid LCE2 148 FGCVQHGIIQHELLHALGFYHEHTRSDRDQYIRVNWQNIIKYFFNNFDKMDTDNLNTRYDYSSVMHYGRTAFGITPQSET 227
Tonguesole LCE 155 FGCINHGIIQHELLHALGFYHEHTRSDRDEYIQINWENINKYFVENFRKMDTDNLNTKYDYTSVMHYGRTAFGTL-RRET 233
Seabass LCE 147 FGCIQQGIIQHELLHALGFYHEHTRSDRDQHIRINWENVNEYFIYNFKKMDTDNLNTPYDYSSIMHYGRTAFGKF-RSET 225
Herring HEa 236 ITPIPNGNVKIGQRQGLSTTDILRINQLYGCCEYDLQTQSGPEMPHSLCLLGKSEL 291
Herring HEb 236 ITPIPNRSVKIGQRQGLSTTDILRINKLYGCCEYDLLHPMVKHRISLRLLK----- 286
Herring HEc 236 ITPIPNGNVQIGQRQGMSTTDILRINKLYGCCKYDLLHPMVKHRISLRLLK----- 286
Herring HEd 235 ITPIPNPNVQIGQRRGLSTTDILRINKLYECCEYDLLHHMVKHHISLRMLK----- 285
Catfish HEa 224 ITPIPDPSVKIGQRNELSAIDILRINTLYKC------------------------- 254
Catfish HEb 224 ITPIPDPSVKIGQRDELSAIDILRINTLYKC------------------------- 254
Cichlid HCE1 235 ISPIPNRNVQIGQRQGMSYWDIRRINLLYGC------------------------- 265
Cichlid HCE2 235 ISPIPNRNVQIGQRQGMSYWDIRRINLLYGC------------------------- 265
Croaker HCE1 235 ITPIPDPNVQIGQRQGMSYWDITRINMLYGC------------------------- 265
Croaker HCE6 231 ITPIPDPNVQIGQRQGMSYWDIMRINMLYGC------------------------- 261
Croaker HCE7 231 ITPIPDPNVQIGQRQGMSYWDIMRINMLYGC------------------------- 261
Seabass HCE 236 ITPIPNANVQIGQRQGMSYWDIMRINLLYSC------------------------- 266
Tonguesole HCE1 232 ITPIPDANVQIGQRRGMSYWDIMRINLLYSC------------------------- 262
Tonguesole HCE2 232 ITPIPDANVQIGQRRGMSYWDIMRINLLYGC------------------------- 262
Tonguesole HCE3 232 ITPIPDANVQIGQRRGMSYWDIMRINLLYGC------------------------- 262
Tonguesole HCE4 232 ITPIPDANVQIGQRRGMSYWDIMRINLLYGC------------------------- 262
Cichlid LCE1 228 LTPIPDPNVPIGQIVGMSDIDILRVNILYQCKK----------------------- 260
Cichlid LCE2 228 LTPIPDPNVPIGQIVGMSDIDILRVNILYQCKK----------------------- 260
Tonguesole LCE 234 ITPIKDPSAPIGQRERVSATDILRINKLYRCW------------------------ 265
Seabass LCE 226 IPIPDPSAPIGQRDSLSIDIDIVRINRLYKCWRYVG-------------------- 261
≥ 50% identity
active sites
cysteine residues

Fig. S1

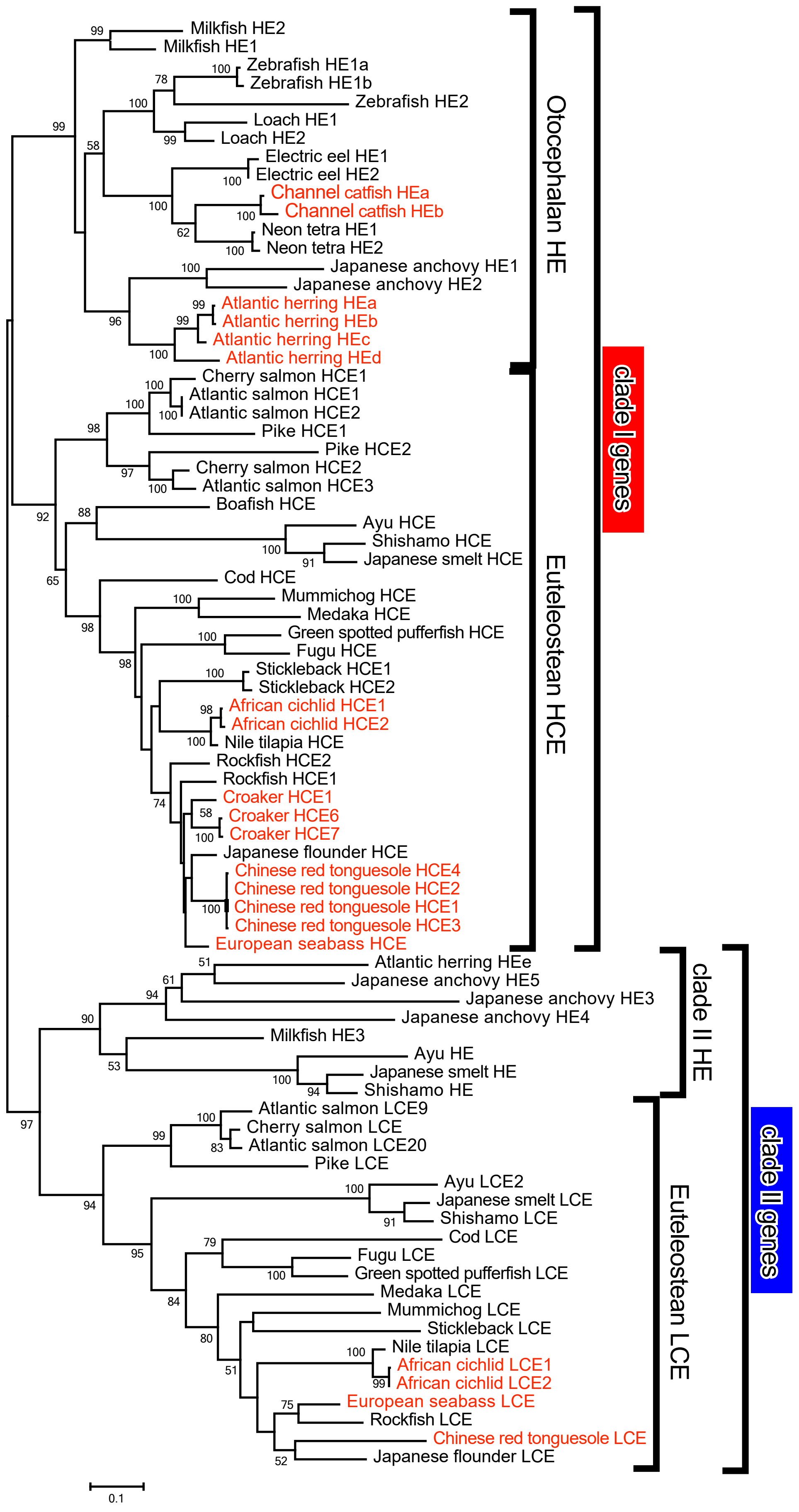
Milkfish HE2
Milkfish HE1
Zebrafish HE1a
Zebrafish HE1b
Zebrafish HE2
Loach HE1
Loach HE2
Electric eel HE1
Electric eel HE2
Channel catfish HEa
Channel catfish HEb
Neon tetra HE1
Neon tetra HE2
Japanese anchovy HE1
Japanese anchovy HE2
Atlantic herring HEa
Atlantic herring HEb
Atlantic herring HEc
Atlantic herring HEd
Cherry salmon HCE1
Atlantic salmon HCE1
Atlantic salmon HCE2
Pike HCE1
Pike HCE2
Cherry salmon HCE2
Atlantic salmon HCE3
Boafish HCE
Ayu HCE
Shishamo HCE
Japanese smelt HCE
Cod HCE
Mummichog HCE
Medaka HCE
Green spotted pufferfish HCE
Fugu HCE
Stickleback HCE1
Stickleback HCE2
African cichlid HCE1
African cichlid HCE2
Nile tilapia HCE
Rockfish HCE2
Rockfish HCE1
Croaker HCE1
Croaker HCE6
Croaker HCE7
Japanese flounder HCE
Chinese red tonguesole HCE4
Chinese red tonguesole HCE2
Chinese red tonguesole HCE1
Chinese red tonguesole HCE3
European seabass HCE
Atlantic herring HEe
Japanese anchovy HE5
Japanese anchovy HE3
Japanese anchovy HE4
Milkfish HE3
Ayu HE
Japanese smelt HE
Shishamo HE
Atlantic salmon LCE9
Cherry salmon LCE
Atlantic salmon LCE20
Pike LCE
Ayu LCE2
Japanese smelt LCE
Shishamo LCE
Cod LCE
Fugu LCE
Green spotted pufferfish LCE
Medaka LCE
Mummichog LCE
Stickleback LCE
Nile tilapia LCE
African cichlid LCE1
African cichlid LCE2
European seabass LCE
Rockfish LCE
Chinese red tonguesole LCE
Japanese flounder LCE
Otocephalan HE
Euteleostean HCE
clade I genes
clade II HE
Euteleostean LCE
clade II genes
0.1

Fig. S2

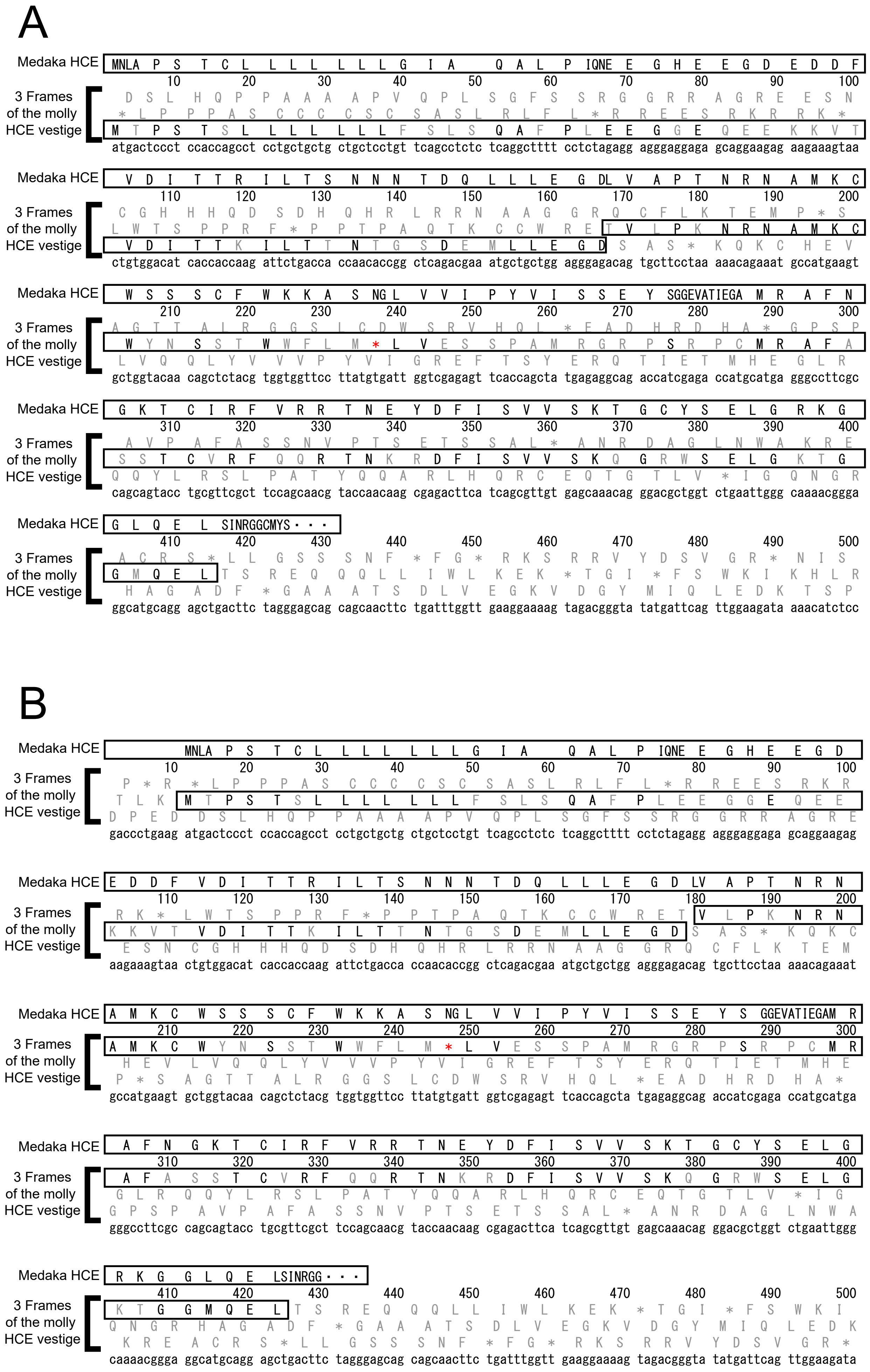
A
Medaka HCE
3 Frames of the molly HCE vestige
atgactccct ccaccagcct cctgctgctg ctgctcctgt tcagcctctc tcaggctttt cctctagagg agggaggaga gcaggaagag aagaaagtaa
ctgtggacat caccaccaag attctgacca ccaacaccgg ctcagacgaa atgctgctgg agggagacag tgcttcctaa aaacagaaat gccatgaagt
gctggtacaa cagctctacg tggtggttcc ttatgtgatt ggtcgagagt tcaccagcta tgagaggcag accatcgaga ccatgcatga gggccttcgc
cagcagtacc tgcgttcgct tccagcaacg taccaacaag cgagacttca tcagcgttgt gagcaaacag ggacgctggt ctgaattggg caaaacggga
ggcatgcagg agctgacttc tagggagcag cagcaacttc tgatttggtt gaaggaaaag tagacgggta tatgattcag ttggaagata aaacatctcc
B
Medaka HCE
3 Frames of the molly HCE vestige
gaccctgaag atgactccct ccaccagcct cctgctgctg ctgctcctgt tcagcctctc tcaggctttt cctctagagg agggaggaga gcaggaagag
aagaaagtaa ctgtggacat caccaccaag attctgacca ccaacaccgg ctcagacgaa atgctgctgg agggagacag tgcttcctaa aaacagaaat
gccatgaagt gctggtacaa cagctctacg tggtggttcc ttatgtgatt ggtcgagagt tcaccagcta tgagaggcag accatcgaga ccatgcatga
gggccttcgc cagcagtacc tgcgttcgct tccagcaacg taccaacaag cgagacttca tcagcgttgt gagcaaacag ggacgctggt ctgaattggg
caaaacggga ggcatgcagg agctgacttc tagggagcag cagcaacttc tgatttggtt gaaggaaaag tagacgggta tatgattcag ttggaagata

Fig. S3

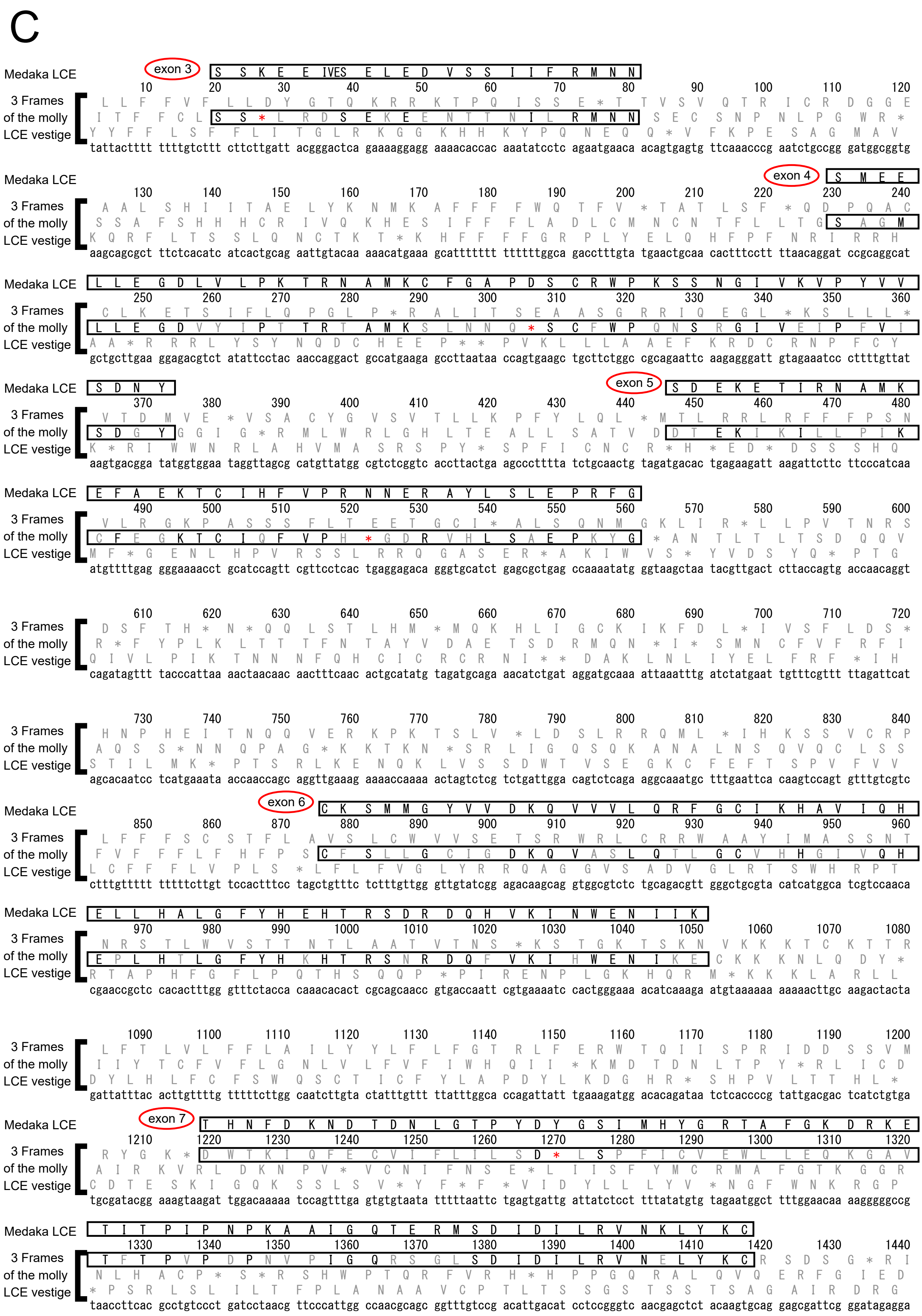
C
Medaka LCE
3 Frames of the molly LCE vestige
exon 3
S S K E E IVES E L E D V S S I I F R M N N
tattactttt ttttgtcttt cttcttgatt acgggactca gaaaaggagg aaaacaccac aaatatcctc agaatgaaca acagtgagtg ttcaaacccg aatctgccgg gatggcggtg
exon 4
S M E E
aagcagcgct ttctcacatc atcactgcag aattgtacaa aaacatgaaa gcattttttt ttttttggca gacctttgta tgaactgcaa cactttcctt ttaacaggat ccgcaggcat
L L E G D L V L P K T R N A M K C F G A P D S C R W P K S S N G I V K V P Y V V
gctgcttgaa ggagacgtct atattcctac aaccaggact gccatgaaga gccttaataa ccagtgaagc tgcttctggc cgcagaattc aagagggatt gtagaaatcc cttttgttat
S D N Y
exon 5
S D E K E T I R N A M K
aagtgacgga tatggtggaa taggttagcg catgttatgg cgtctcggtc acttactga agccctttta tctgcaactg tagatgacac tgagaagatt aagattcttc ttcccatcaa
E F A E K T C I H F V P R N N E R A Y L S L E P R F G
atgttttgag gggaaaacct gcatccagtt cgttcctcac tgaggagaca gggtgcatct gagcgctgag ccaaaatatg ggtaagctaa tacgttgact cttaccagtg accaacaggt
cagatagttt tacccattaa aactaacaac aactttcaac actgcatatg tagatgcaga aacatctgat aggatgcaaa attaaatttg atctatgaat tgtttcgttt ttagattcat
agcacaatcc tcatgaaata accaaccagc aggttgaaag aaaaccaaaa actagtctcg tctgattgga cagtctcaga aggcaaatgc tttgaattca caagtccagt gtttgtcgtc
exon 6
C K S M M G Y V V D K Q V V V L Q R F G C I K H A V I Q H
ctttgttttt tttttcttgt tccactttcc tagctgtttc tctttgttgg gttgtatcgg agacaagcag gtggcgtctc tgcagacgtt gggctgcgta catcatggca tcgtccaaca
E L L H A L G F Y H E H T R S D R D Q H V K I N W E N I I K
cgaaccgctc cacactttgg gtttctacca caaacacact cgcagcaacc gtgaccaatt cgtgaaaatc cactgggaaa acatcaaaga atgtaaaaaa aaaaacttgc aagactacta
gattatttac acttgttttg tttttcttgg caatcttgta ctatttgttt ttatttggca ccagattatt tgaaagatgg acacagataa tctcaccccg tattgacgac tcatctgtga
exon 7
T H N F D K N D T D N L G T P Y D Y G S I M H Y G R T A F G K D R K E
tgcgatacgg aaagtaagat tggacaaaaa tccagtttga gtgtgtaata tttttaattc tgagtgattg attatctcct tttatatgtg tagaatggct tttggaacaa aagggggccg
T I T P I P N P K A I G Q T E R M S D I D I L R V N K L Y K C
taaccttcac gcctgtccct gatcctaacg ttcccattgg ccaacgcagc ggtttgtccg acattgacat cctccggtc aacgagctct acaagtgcag gagcgattcg ggatagagga

Fig. S3

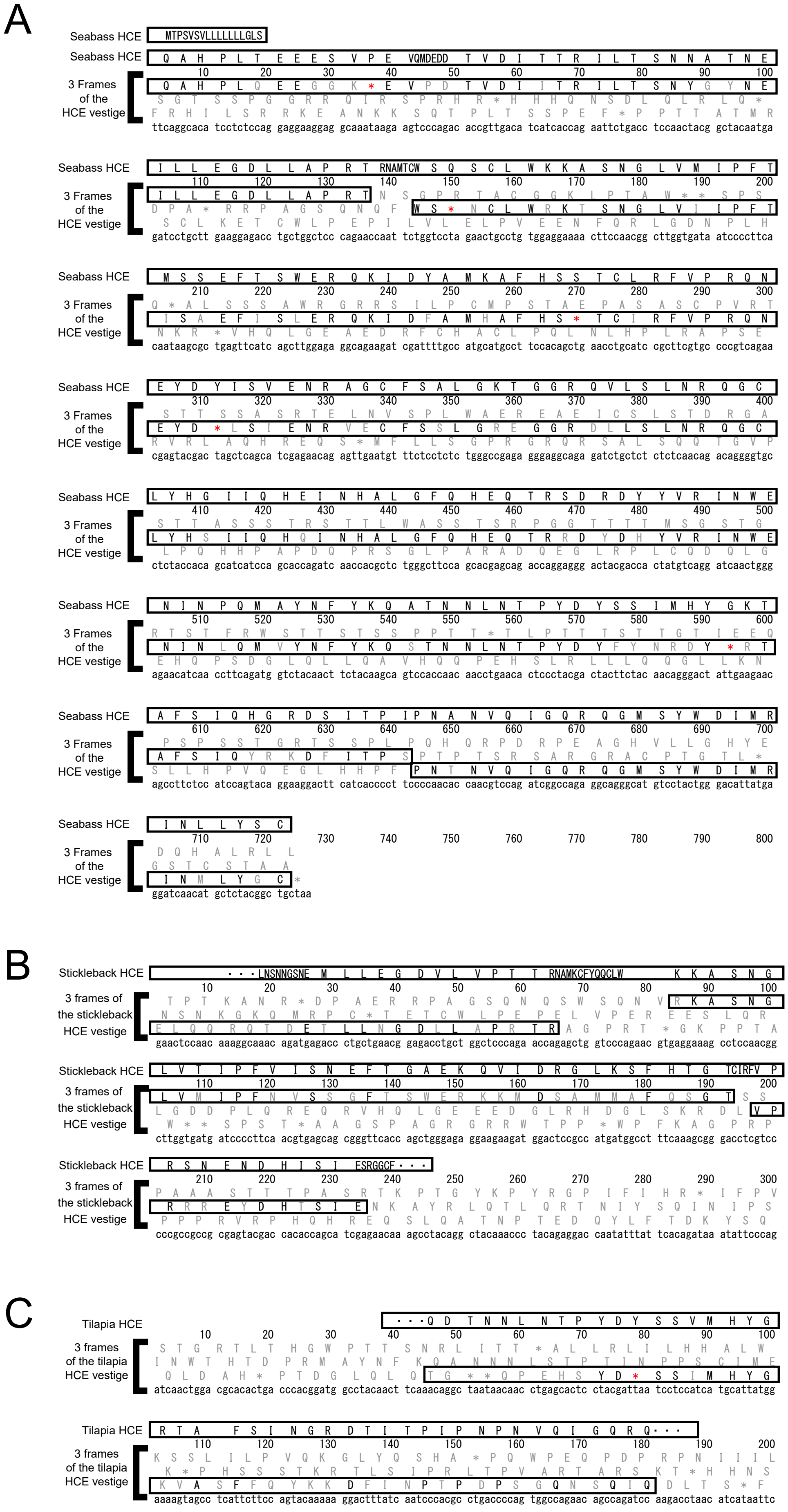
A
Seabass HCE
3 Frames of the HCE vestige
B
Stickleback HCE
3 frames of the stickleback HCE vestige
C
Tilapia HCE
3 frames of the tilapia HCE vestige

Fig. S4

A

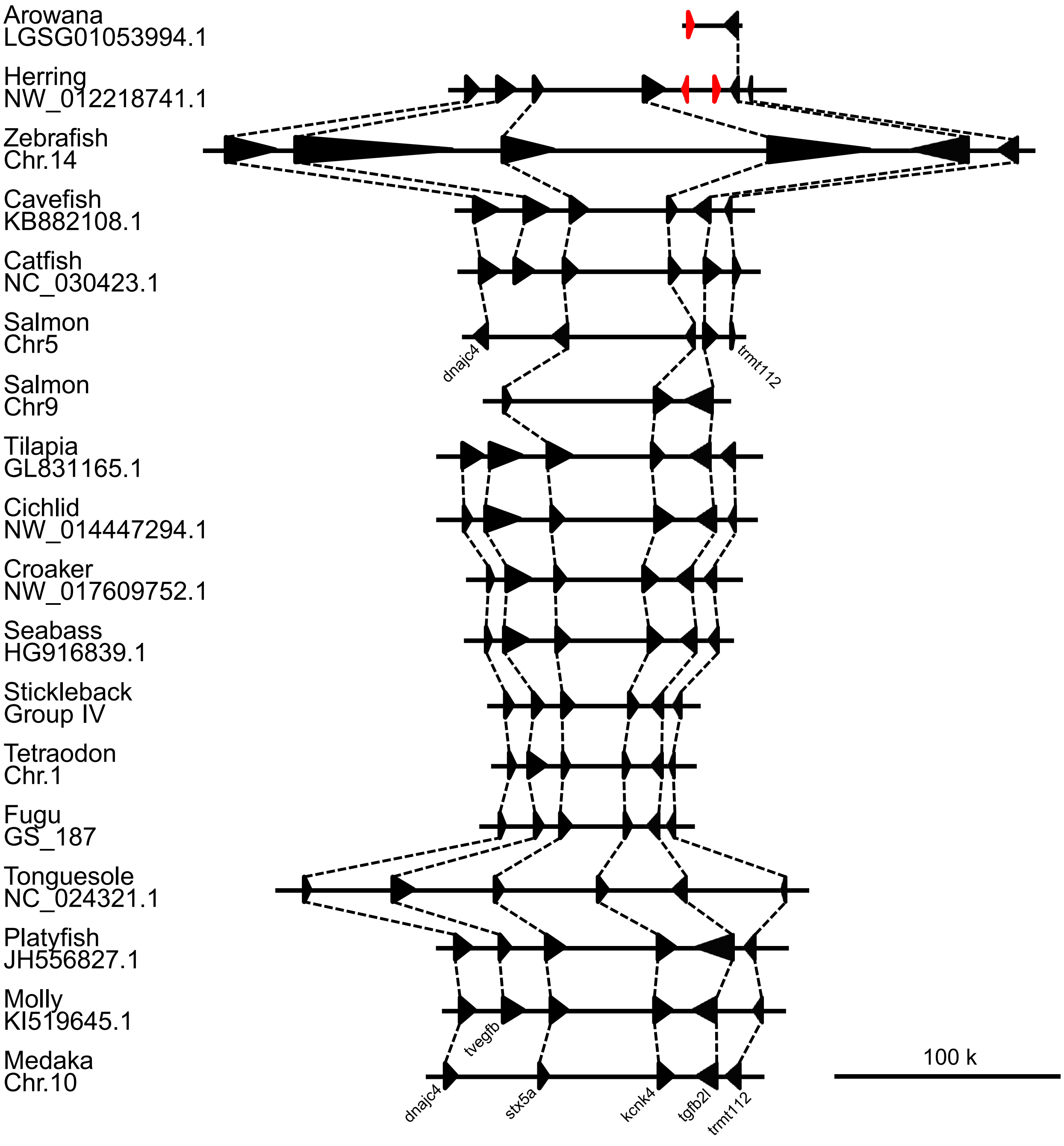

Arowana
LGSG01053994.1
Herring
NW_012218741.1
Zebrafish
Chr.14
Cavefish
KB882108.1
Catfish
NC_030423.1
Salmon
Chr5
dnajc4
trmt112
Salmon
Chr9
Tilapia
GL831165.1
Cichlid
NW_014447294.1
Croaker
NW_017609752.1
Seabass
HG916839.1
Stickleback
Group IV
Tetraodon
Chr.1
Fugu
GS_187
Tonguesole
NC_024321.1
Platyfish
JH556827.1
Molly
KI519645.1
tvegfb
Medaka
Chr.10
100 k
dnajc4
stx5a
kcnk4
tgfb2l
trmt112

Fig. S5

B

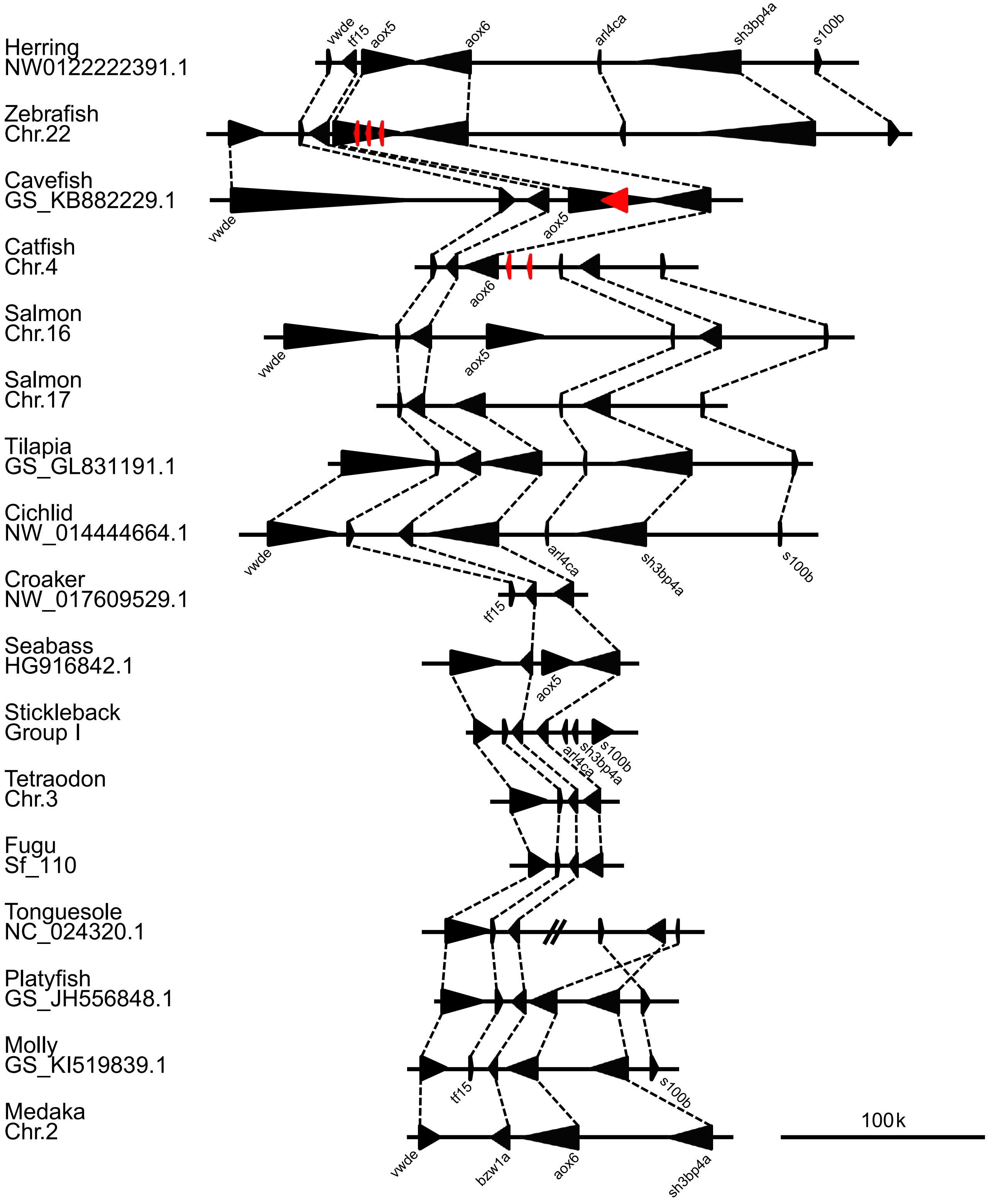
Herring
NW0122222391.1
Zebrafish
Chr.22
Cavefish
GS_KB882229.1
Catfish
Chr.4
Salmon
Chr.16
Salmon
Chr.17
Tilapia
GS_GL831191.1
Cichlid
NW_014444664.1
Croaker
NW_017609529.1
Seabass
HG916842.1
Stickleback
Group I
Tetraodon
Chr.3
Fugu
Sf_110
Tonguesole
NC_024320.1
Platyfish
GS_JH556848.1
Molly
GS_KI519839.1
Medaka
Chr.2
vwde
tf15
aox5
aox6
arl4ca
sh3bp4a
s100b
bzw1a
100k

Fig. S5

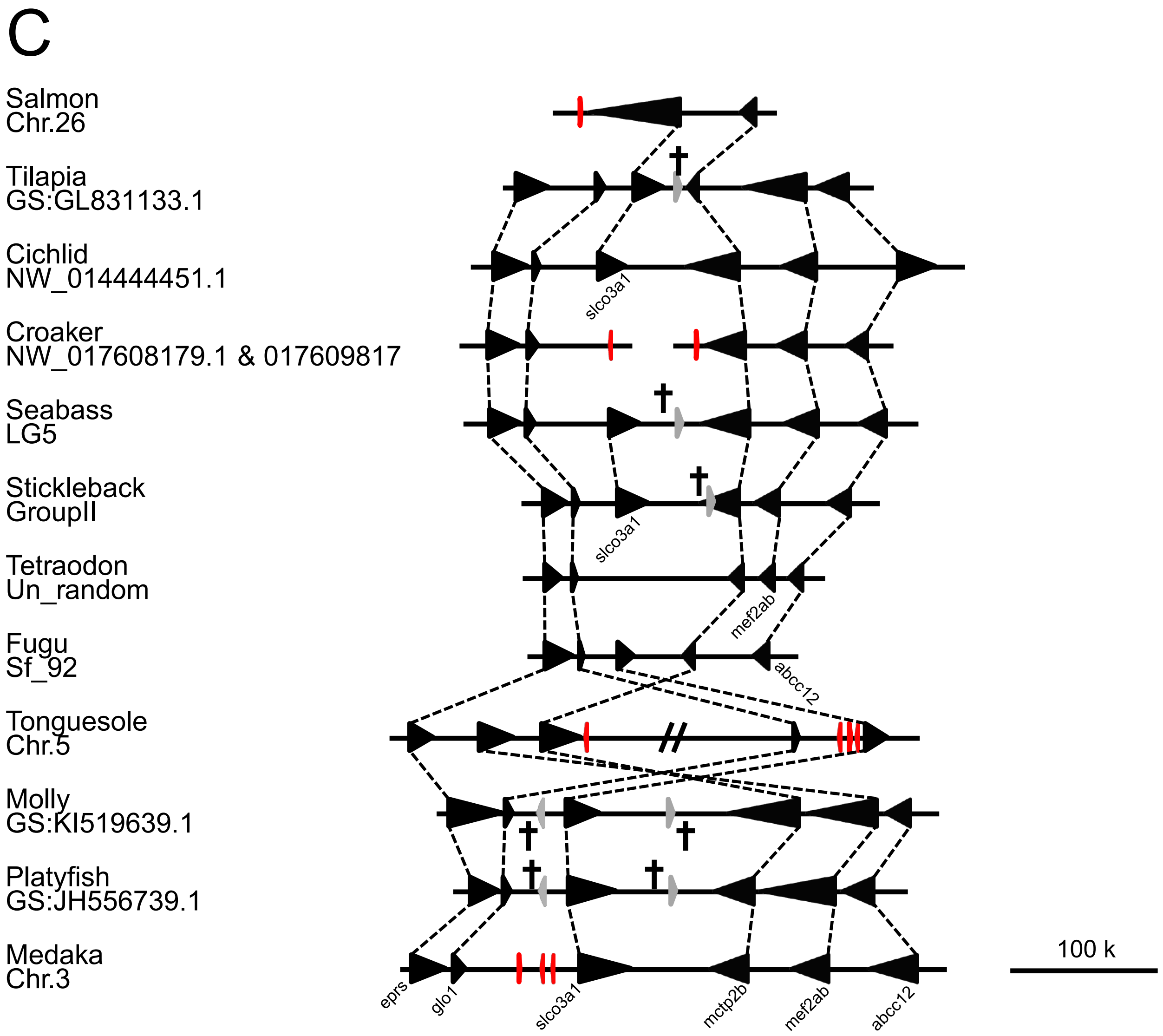
C
Salmon
Chr.26
Tilapia
GS:GL831133.1
Cichlid
NW_014444451.1
Croaker
NW_017608179.1 & 017609817
Seabass
LG5
Stickleback
GroupII
Tetraodon
Un_random
Fugu
Sf_92
Tonguesole
Chr.5
Molly
GS:KI519639.1
Platyfish
GS:JH556739.1
Medaka
Chr.3
slco3a1
slco3a1
mef2ab
abcc12
eprs
glo1
slco3a1
mctp2b
mef2ab
abcc12
100 k

Fig. S5

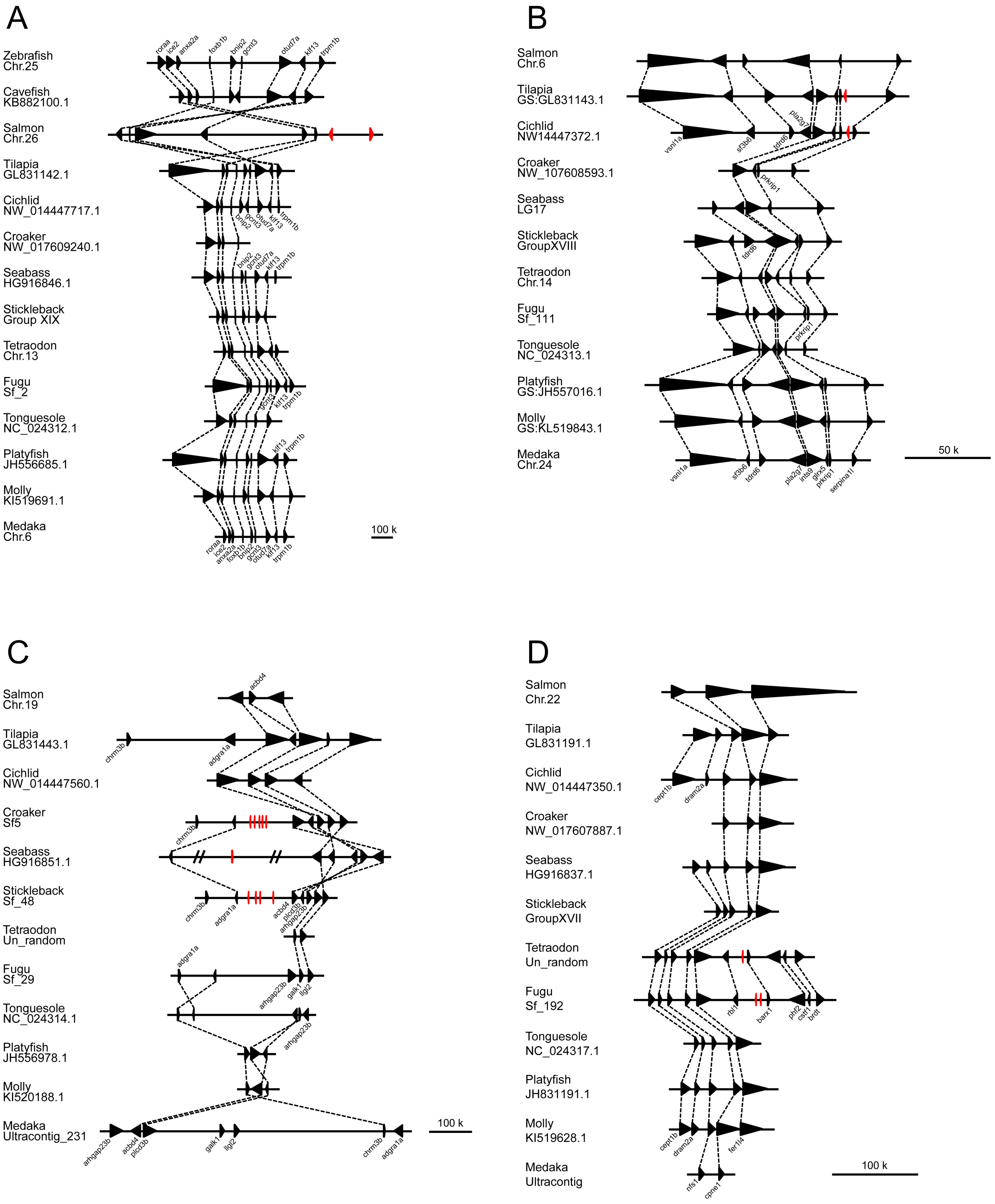
A
Zebrafish Chr.25
Cavefish KB882100.1
Salmon Chr.26
Tilapia GL831142.1
Cichlid NW_014447717.1
Croaker NW_017609240.1
Seabass HG916846.1
Stickleback Group XIX
Tetraodon Chr.13
Fugu Sf_2
Tonguesole NC_024312.1
Platyfish JH556685.1
Molly KI519691.1
Medaka Chr.6
100 k
B
Salmon Chr.6
Tilapia GS:GL831143.1
Cichlid NW14447372.1
Croaker NW_107608593.1
Seabass LG17
Stickleback GroupXVIII
Tetraodon Chr.14
Fugu Sf_111
Tonguesole NC_024313.1
Platyfish GS:JH557016.1
Molly GS:KL519843.1
Medaka Chr.24
50 k
C
Salmon Chr.19
Tilapia GL831443.1
Cichlid NW_014447560.1
Croaker Sf5
Seabass HG916851.1
Stickleback Sf_48
Tetraodon Un_random
Fugu Sf_29
Tonguesole NC_024314.1
Platyfish JH556978.1
Molly KI520188.1
Medaka Ultracontig_231
100 k
D
Salmon Chr.22
Tilapia GL831191.1
Cichlid NW_014447350.1
Croaker NW_017607887.1
Seabass HG916837.1
Stickleback GroupXVII
Tetraodon Un_random
Fugu Sf_192
Tonguesole NC_024317.1
Platyfish JH831191.1
Molly KI519628.1
Medaka Ultracontig
100 k

Fig. S6

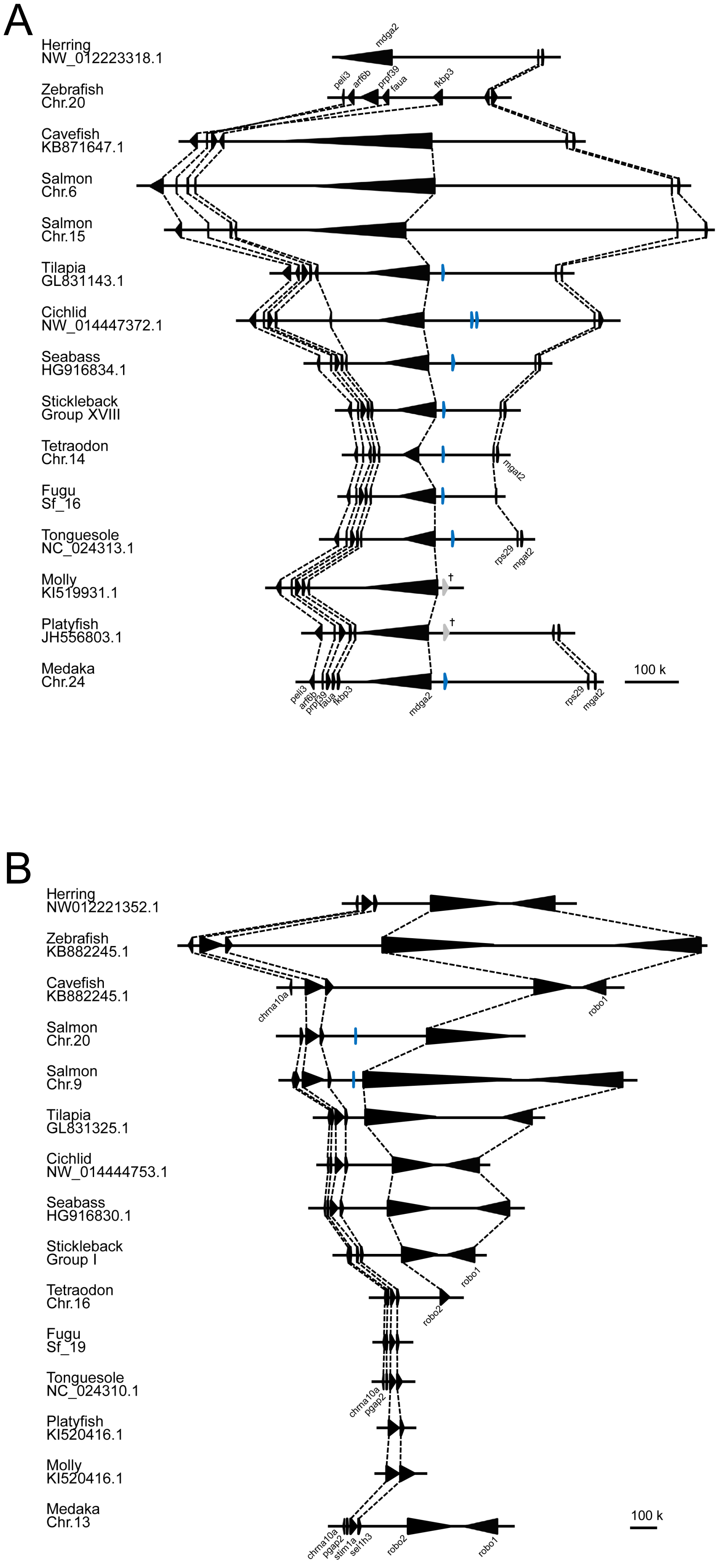

A
Herring
NW_012223318.1
Zebrafish
Chr.20
Cavefish
KB871647.1
Salmon
Chr.6
Salmon
Chr.15
Tilapia
GL831143.1
Cichlid
NW_014447372.1
Seabass
HG916834.1
Stickleback
Group XVIII
Tetraodon
Chr.14
Fugu
Sf_16
Tonguesole
NC_024313.1
Molly
KI519931.1
Platyfish
JH556803.1
Medaka
Chr.24
mdga2
peli3
arf6b
prpf39
faua
fkbp3
mgat2
rps29
100 k
B
Herring
NW012221352.1
Zebrafish
KB882245.1
Cavefish
KB882245.1
Salmon
Chr.20
Salmon
Chr.9
Tilapia
GL831325.1
Cichlid
NW_014444753.1
Seabass
HG916830.1
Stickleback
Group I
Tetraodon
Chr.16
Fugu
Sf_19
Tonguesole
NC_024310.1
Platyfish
KI520416.1
Molly
KI520416.1
Medaka
Chr.13
chrna10a
robo1
robo2
pgap2
stim1a
sel1h3
100 k

Fig. S7

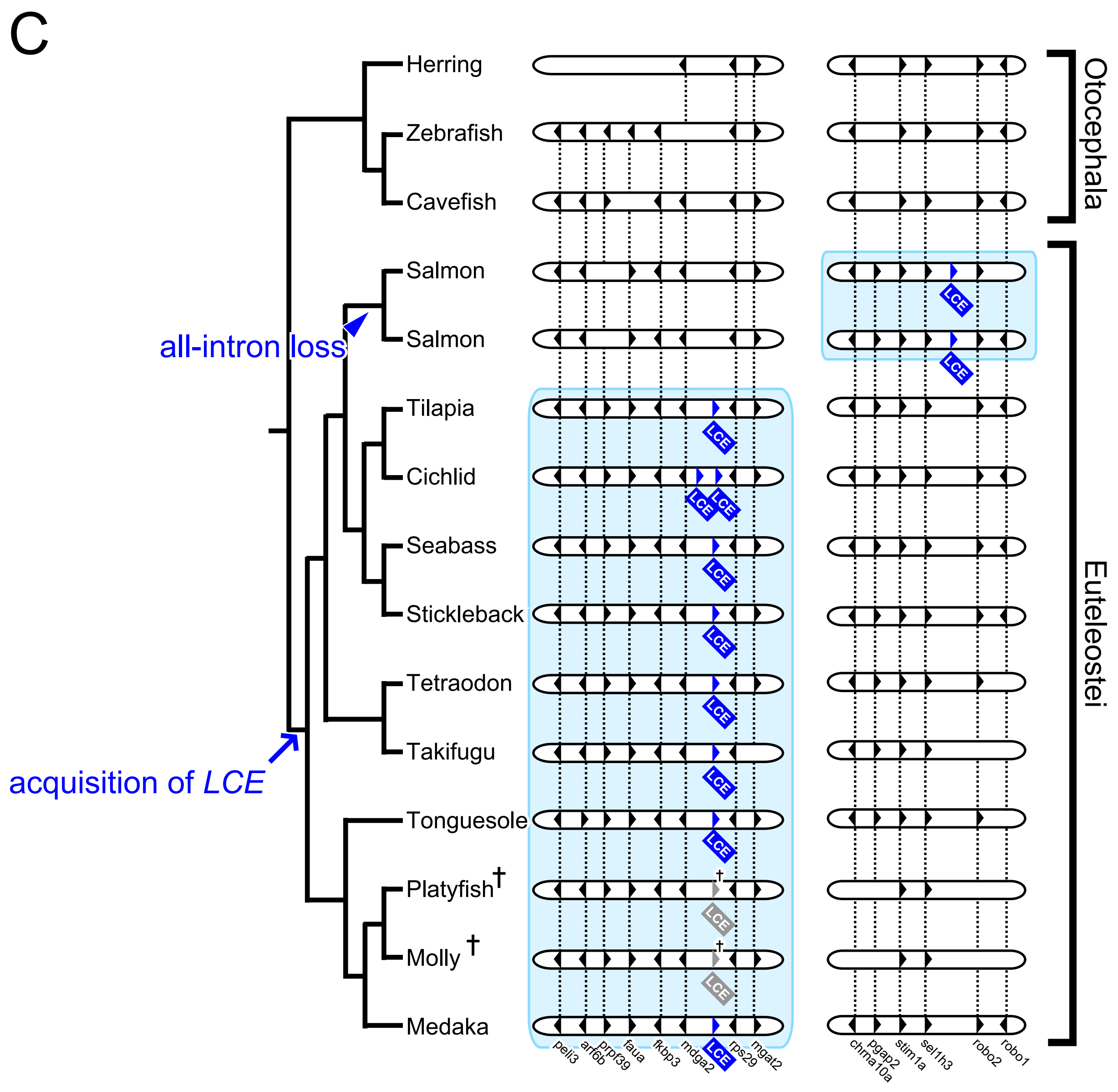

C
Herring
Zebrafish
Cavefish
Salmon
Salmon
Tilapia
Cichlid
Seabass
Stickleback
Tetraodon
Takifugu
Tonguesole
Platyfish†
Molly †
Medaka
all-intron loss
acquisition of LCE
Otocephala
Euteleostei
LCE
peli3
arf6b
prpf39
faua
fkbp3
mdga2
rps29
mgat2
chrna10a
pgap2
stim1a
sel1h3
robo2
robo1

Fig. S7

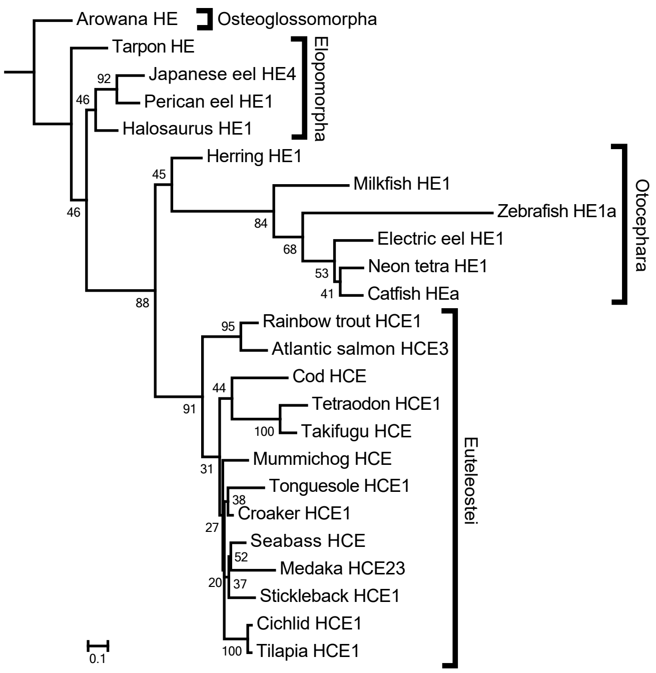
Arowana HE
Osteoglossomorpha
Tarpon HE
Japanese eel HE4
Perican eel HE1
Halosaurus HE1
Elopomorpha
Herring HE1
Milkfish HE1
Zebrafish HE1a
Electric eel HE1
Neon tetra HE1
Catfish HEa
Otocephara
Rainbow trout HCE1
Atlantic salmon HCE3
Cod HCE
Tetraodon HCE1
Takifugu HCE
Mummichog HCE
Tonguesole HCE1
Croaker HCE1
Seabass HCE
Medaka HCE23
Stickleback HCE1
Cichlid HCE1
Tilapia HCE1
Euteleostei
92
46
46
45
84
68
53
41
88
95
44
100
91
31
38
27
52
20
37
100
0.1

Fig. S8

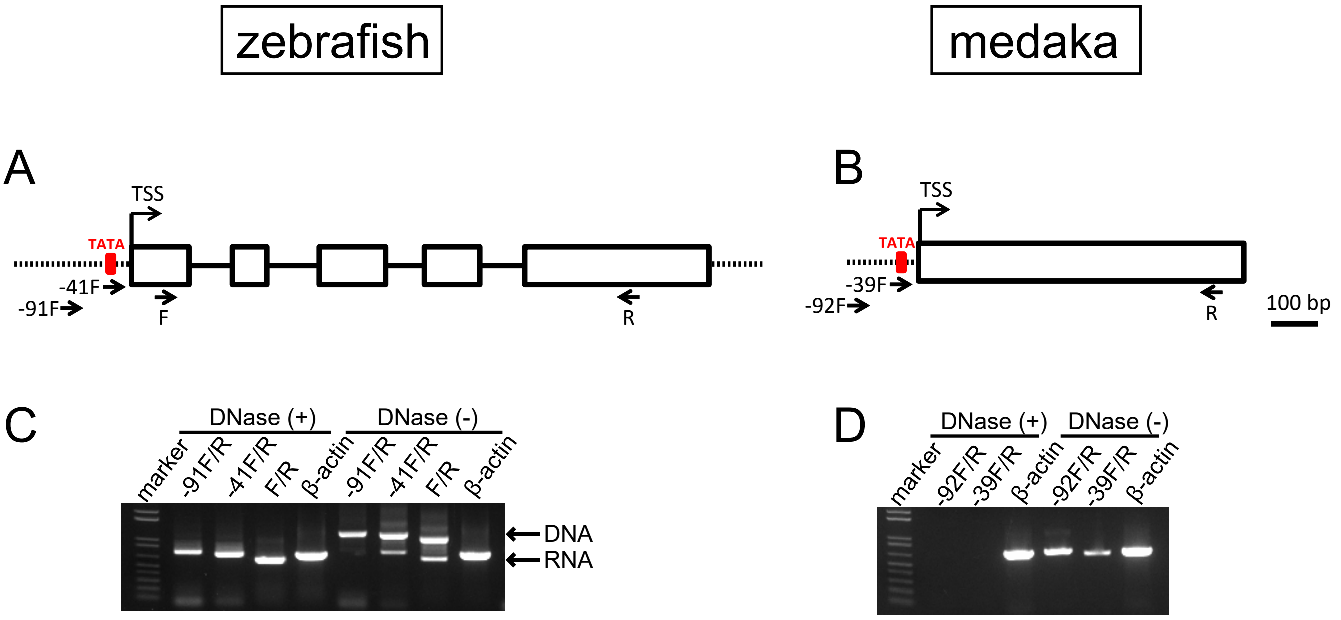
zebrafish
medaka
A
TSS
TATA
-41F
-91F
F
R
B
TSS
TATA
-39F
-92F
R
100 bp
C
DNase (+)
DNase (-)
marker
-91F/R
-41F/R
F/R
β-actin
-91F/R
-41F/R
F/R
β-actin
DNA
RNA
D
DNase (+)
DNase (-)
marker
-92F/R
-39F/R
β-actin
-92F/R
-39F/R
β-actin

Fig. S9

Table S1. The accession numbers of hatching enzyme genes from Fig. S2

common name	scientific name	gene name	accession number
Milkfish	Chanos chanos	HE1	AB48009.1
		HE2	AB48010.1
		HE3	AB48011.1
Japanese anchovy	Engraulis japonicus	HE1	AB433587.1
		HE2	AB433588.1
		HE3	AB433589.1
		HE4	AB433590.1
		HE5	AB433591.1
Atlantic herring	Clupea herengus	HEa	LC337369
		HEb	LC337370
		HEc	LC337371
		HEd	LC337372
Loach	Misgurnus anguillicaudatus	HE1	AB480012.1
		HE2	AB480013.1
Zebrafish	Danio rerio	HE1a	NM_001045174.2
		HE1b	NM_213635.2
		HE2	NM_001098188.1
Channeal catfish	Ictalurus panctatsu	HEa	LC337373
		HEb	LC337374
Neon tetra	Paracheirodon innesi	HE1	AB480014.1
		HE2	AB480015.1
Electric eel	Electrophorus electricus	HE1	AB480019.1
		HE2	AB480020.1
Pike	Esox americanus	HCE1	AB480025.1
		HCE2	AB480026.1
		LCE	AB480027.1
Cherry salmon	Oncorhyncus masou	HCE1	AB175619.1
		HCE2	AB175618.1
		LCE	AB480021.1
Atlantic salmon	Salmo salar	HCE1	XM_014176676.1
		HCE2	XM_014176676.1
		HCE3	XM_014176785.1
		LCE9	XM_014176676.1
		LCE20	NM_00163081.1
Ayu	Plecoglossus altivelis	HCE	AB256940.1
		LCE2	AB256942.1
		HE	AB256941.1
Shishamo	Spirinchus lanceolatus	HCE	AB549216.1
		LCE	AB549217.1
		HE	AB549218.1
Japanese smelt	Hypomesus nipponensis	HCE	AB549219.1
		LCE	AB549220.1
		HE	AB549221.1
Boafish	Stomias nebulosus	HCE	AB480028.1
Cod	Gadus macrocephalus	HCE	AB480029.1
		LCE	AB480030.1
Green spotted pufferfish	Tetraodon nigroviridis	HCE	AB246043.1
		LCE	AB246044.1
Fugu	Takifugu ruburipes	HCE	NM_001078638.2
		LCE	NM_001078597.1
Stickleback	Gasterosteus aculeatus	HCE1	NM_001267673.1
		HCE2	AB353109.1
		LCE	NM_001267674.1
Nile tilapia	Oreochromis niloticus	HCE	XM_005449826.3
		LCE	XM_019346261.1
African cichlid	Maylandia zebra	HCE1	LC337375
		HCE2	LC337376
		LCE1	LC337377
		LCE2	LC337378
Rockfish	Helicolenus hilgendorfi	HCE1	AB353102.1
		HCE2	AB353103.1
		LCE	AB353104.1
European seabass	Dicentrarchus labrax	HCE	LC337379
		LCE	LC337380
Japanese flounder	Paralichthys olivaceus	HCE	AB480031.1
		LCE	AB480032.1
Chinese red tonguesole	Cynoglossus semilaevis	HCE1	LC337381
		HCE2	LC337382
		HCE3	LC337383
		HCE4	LC337384
		LCE	LC337385
Croaker	Larimichthys crocea	HCE1	LC337386
		HCE6	LC337387
		HCE7	LC337388
Mummichog	Fundulus heteroclitus	HCE	NM_001309926.1
		LCE	NM_001309911.1
Medaka	Oryzias latipes	HCE	NM_001201498.1
		LCE	NM_001104822.1
